# Single-cell and spatial transcriptomics map lung-adaptive metastasis programs through Exoc4, Cd36–vasculogenic mimicry, and Cxcr2 signaling in triple-negative breast cancer

**DOI:** 10.1038/s41419-026-09049-6

**Published:** 2026-07-07

**Authors:** Hsiao-Chen Lee, Chia-Yu Kuo, Fang-Ming Chen, Ming-Feng Hou, Hung-Pei Tsai, Yu-Yuan Wu, Kai-Chien Chuang, Ling-Yu Wu, Sheng-Feng Pan, Ying-Ming Tsai, Ya-Ling Hsu

**Affiliations:** 1 Division of Plastic Surgery, Department of Surgery, Kaohsiung Municipal Siaogang Hospital, Kaohsiung Medical University Hospital, Kaohsiung Medical University, Kaohsiung, Taiwan, ROC; 2 Division of Breast Oncology and Surgery, Department of Surgery, Kaohsiung Medical University Hospital, Kaohsiung, Taiwan, ROC; 3 Graduate Institute of Clinical Medicine, Kaohsiung Medical University, Kaohsiung, Taiwan, ROC; 4 Division of Neurosurgery, Department of Surgery, Kaohsiung Medical University Hospital, Kaohsiung Medical University, Kaohsiung, Taiwan, ROC; 5 Graduate Institute of Medicine, College of Medicine, Kaohsiung Medical University, Kaohsiung, Taiwan, ROC; 6 Division of Pulmonary and Critical Care Medicine, Kaohsiung Medical University Hospital, Kaohsiung Medical University, Kaohsiung, Taiwan, ROC; 7 Drug Development and Value Creation Research Center, Kaohsiung Medical University, Kaohsiung, Taiwan, ROC; 8 School of Medicine, College of Medicine, Kaohsiung Medical University, Kaohsiung, Taiwan, ROC; 9 Division of Pulmonary Medicine, Kaohsiung Medical University Gangshan Hospital, Kaohsiung Medical University, Kaohsiung, Taiwan, ROC; 10 Center for Disease Multi-Omics Research, Kaohsiung Medical University, Kaohsiung, Taiwan, ROC

**Keywords:** Tumour heterogeneity, Cancer microenvironment

## Abstract

Triple-negative breast cancer (TNBC) is an aggressive subtype associated with high metastatic potential and poor outcomes, with lung metastasis representing one of the most frequent and life-threatening events. However, the mechanism how cellular plasticity and reprogramming that enable TNBC cells to colonize in the lung remain incompletely understood. Here, we integrated single-cell RNA sequencing and spatial transcriptomics on paired MMTV-PyMT primary mammary tumors and lung metastatic tumors, complemented by functional assays in 4T1 mouse models, to delineate the cellular heterogeneity and molecular mechanisms driving TNBC lung metastasis. We identified three metastasis-associated subtypes—C12-Cd, Cdh5-like, and Wfdc12—each enriched in metastatic lesions and exhibiting distinct biological programs. The C12-Cd subcluster was characterized by the upregulation of Exoc4, a vesicle-trafficking regulator associated with a poor prognosis in patients with TNBC. The Cdh5-like cluster displayed a hypoxia-driven vascular phenotype, in which Hif-2α–mediated Cd36 expression promoted vasculogenic mimicry and metastatic colonization. The Wfdc12 cluster secreted Cxcr2 ligands (Cxcl1/2) and Cxcr2 blockade significantly reduced lung metastasis in vivo, underscoring therapeutic potential. Notably, cross-subtypes of lung metastatic TNBC analysis revealed *Sftpc* upregulation, reflecting organ-adaptive transcriptional reprogramming that may facilitate tumor cell survival in the pulmonary niche. Collectively, our findings provide a comprehensive cellular and molecular atlas of TNBC lung metastasis. We establish Exoc4, the Hif-2α–Cd36 vasculogenic mimicry axis, Cxcrl1/2–Cxcr2 signaling, and Sftpc as critical mediators of metastatic adaptation. These results not only bridge key knowledge gaps in metastatic heterogeneity and organ-specific adaptation but also highlight novel prognostic markers and therapeutic vulnerabilities with translational potential for treating TNBC lung metastasis.

## Introduction

Triple-negative breast cancer (TNBC) is an aggressive breast cancer subtype that lacks expression of estrogen receptor (ER), progesterone receptor (PR), and human epidermal growth factor receptor 2 (Her2), accounting for approximately 15–20% of all breast cancers [1]. TNBC is associated with early recurrence, high metastatic potential, and limited therapeutic options, resulting in poor clinical outcomes compared to other breast cancer subtypes [2]. Despite advances in chemotherapy and immunotherapy, the prognosis of patients with metastatic TNBC remains dismal, with lung metastasis being one of the most frequent and life-threatening manifestations [3, 4]. The lung microenvironment provides a unique niche that supports metastatic outgrowth, but the cellular and molecular programs that enable TNBC cells to colonize and adapt to the lung remain incompletely understood. To declinate understanding the mechanisms that drive TNBC metastasize to lung is therefore a pressing unmet need, not only to advance biological knowledge, but also to identify novel therapeutic strategies that may improve patient survival.

Breast cancer progression is increasingly recognized as a highly heterogeneous process. Intra-tumoral heterogeneity, defined by the coexistence of genetically and phenotypically distinct subclones, plays a fundamental role in tumor evolution, therapy resistance, and metastatic dissemination [5, 6]. In TNBC, this heterogeneity is particularly pronounced and represents a major determinant of its aggressive clinical behavior [7]. Increasing evidence suggests that metastatic tumor cells do not simply retain the genomic or phenotypic characteristics of their primary-site counterparts, but instead undergo profound transcriptional and phenotypic reprogramming, thereby acquiring traits that facilitate adaptation within the metastatic niche [8]. Brain metastases of TNBC exhibit altered expression of DNA repair–related genes in animal models [9]. More broadly, metastatic lesions often display distinct metabolic dependencies, extracellular matrix (ECM) remodeling, vascular remodeling, immune evasion mechanisms, and organ-specific gene expression programs that are rarely observed in primary tumors [10, 11]. This complexity is further compounded by the tendency of metastatic lesions to develop resistance to anti-angiogenic therapies, immunotherapies, and chemotherapies [12, 13]. Together, these observations underscore the urgent need to map the precise cellular subpopulations and molecular programs that orchestrate TNBC metastasis to the lungs. However, the critical subclusters and their defining features that specifically govern TNBC adaptation to the lung remain poorly characterized.

To address these challenges, we applied high-resolution transcriptomic approaches to systematically dissect the cellular heterogeneity and spatial architecture of TNBC primary tumors and their matched lung metastases [14]. This enabled us to resolve metastasis-associated subtypes, reconstruct lineage trajectories, and identify spatially confined transcriptional programs that underlie metastatic adaptation. We uncovered distinct molecular programs—including vesicle trafficking mediated by Exoc4, hypoxia-driven vasculogenic mimicry through the Hif-2α–Cd36 axis, and chemokine-mediated dissemination via Cxcl1/2–Cxr2 signaling—that collectively shape lung colonization. Moreover, we identified Sftpc as a lung-specific marker of metastatic TNBC, reflecting organ-adaptive transcriptional reprogramming. Together, our study provides a comprehensive atlas of TNBC lung metastasis and highlights novel prognostic markers and therapeutic vulnerabilities with translational potential.

## Methods

### Mouse models

Female BALB/c mice (6–8 weeks old) were purchased from the National Laboratory Animal Center, Taiwan and maintained under the specific pathogen–free condition. Lung metastatic models were generated by using 4T1 cells orthotopic model (*n* = 3) or MMTV-PyMT transgenic mice (*n* = 3) that spontaneously develop mammary tumors and lung metastases. All animal procedures were approved by the Institutional Animal Care and Use Committee of Kaohsiung Medical University (KMU, 112119). Orthotopic breast cancer models were established by injecting 1 × 10⁶ 4T1 cells into the mammary fat pad. For neutralizing antibody treatment, mice injected with 4T1 cells were treated with anti-Cxcr2 neutralizing antibody (10 mg/kg, i.p., Catalog #: MAB2164, R&D system, *n* = 10) or isotype control (10 mg/kg, i.p., twice weekly, *n* = 8). Lung metastases were evaluated after 4 weeks by histological counting of metastatic nodules. Cd36-overexpressing or control 4T1-P cells, as well as Cd36- or Sftpc-knockdown 4T1-L cells, were labeled with PKH26 dye (Sigma-Aldrich, St. Louis, MO) according to the manufacturer’s instructions and injected into mice via the tail vein (*n* = 8 per group) via the tail vein. Lungs were harvested at 24 h and 7 days post-injection, embedded in OCT (Optimal Cutting Temperature) compound (Sakura Finetek, Torrance, CA), and cryosectioned at a thickness of 5 µm. Sections were examined by a confocal fluorescence microscopy (Zeiss, Oberkochen, Germany) to detect PKH26-labeled tumor cells and assess early colonization.

### Sample collection and preparation

Primary mammary tumors and matched lung metastatic nodules (*n* = 3) were dissected from MMTV-PyMT transgenic mice, while lung metastatic tumor nodules were obtained from the 4T1 spontaneous metastasis mouse model. Tumor tissues were mechanically and enzymatically dissociated into single-cell suspensions using the Tumor and Lung Dissociation Kit (Miltenyi Biotec, Bergisch Gladbach, Germany) according to the manufacturer’s protocol. After dissociation, cell suspensions were filtered through a 40-μm cell strainer, and red blood cells were removed using RBC lysis buffer. Cell viability was assessed by trypan blue staining, and only samples with viability >90% were used for downstream single-cell RNA sequencing. EpCAM⁺ epithelial tumor cells were enriched by magnetic-activated cell sorting (MACS; Miltenyi Biotec). Mycoplasma testing was routinely performed every three months.

### Flowcytometry analysis

Cells were stained with fluorochrome-conjugated antibodies against Cd36, EpCAM (Cd326), and Cd31 according to the manufacturers’ instructions, and analyzed by flow cytometry. Cell purity was consistently greater than 90%, as determined by flow cytometry using anti-EpCAM and anti-Cd31 antibodies, with Cd31 serving as a marker to assess endothelial cell contamination (Supplementary Fig. 1). Cd36 expression in cells was quantified as mean fluorescence intensity (MFI).

### Single-cell RNA sequencing (scRNA-seq) and Spatial transcriptomics (ST)

Single-cell suspensions were loaded onto a 10x Genomics Chromium platform to generate single-cell Gel Bead-in-Emulsions. Libraries were prepared using the Chromium Single Cell 3’ v3 kit (10x Genomics, Pleasanton, CA) and sequenced on an Illumina NovaSeq 6000 (Illumina, San Diego, CA). Raw sequencing data were processed with Cell Ranger (v7.1.0, 10x Genomics), and downstream analyses were performed using Scanpy (v1.10.2; https://scanpy.readthedocs.io/). Low-quality cells (> five-fold median absolute deviations (MAD) for log1p_total_counts, log1p_n_genes_by_counts, pct_counts_in_top_20_genes 15 or >15% mitochondrial reads) were excluded. Doublets were predicted and removed using SOLO from scvi-tools (v1.1.6; https://scvi-tools.org/). Normalization, dimensionality reduction, and clustering were conducted using PCA (Principal Component Analysis) and UMAP (Uniform Manifold Approximation and Projection), followed by identification of cancer cell subclusters based on differential expression of canonical markers and reference to the CellMarker 2.0 database (http://bio-bigdata.hrbmu.edu.cn/CellMarker/). Differentially expressed gene (DEG) analysis was performed using Loupe Browser (v8.0, 10x Genomics). Genes with a fold change (FC) greater than 2 and a *p*-value < 0.05 were considered statistically significant. Only genes detected in at least 20% of cells in either cluster or group were retained for downstream analysis. Mitochondrial, ribosomal, and hemoglobin-related genes were excluded from downstream DEG interpretation. The experimental procedures and bioinformatics analyses for spatial transcriptomics are described in our previous publication [14].

### Bioinformatics

Gene Ontology and KEGG pathway enrichment analyses were performed using the DAVID database (https://david.ncifcrf.gov/). CytoTRACE (v0.3.3; https://cytotrace.stanford.edu/) was applied to estimate cellular differentiation potential. Trajectory inference was conducted using Monocle 2 (v2.22; http://cole-trapnell-lab.github.io/monocle-release/) to reconstruct lineage relationships among tumor subclusters. Cell–cell interaction analysis was performed using CellChat (https://github.com/jinworks/CellChat) to infer intercellular communication networks among different cell populations.

### Gene and protein expression-based survival analysis

The prognostic significance of specific genes in relation to overall survival (OS) was assessed using the KM plotter online tool based on the RNA-seq dataset (https://kmplot.com/analysis/index.php?p=home). Patients were divided into high- and low-expression groups using the default cutoff settings provided by the platform, and Kaplan–Meier survival curves were generated. The hazard ratio (HR), 95% confidence interval (CI), and log-rank *p* value were used to evaluate the association between gene expression and patient survival. In parallel, the association between the expression levels of specific proteins and OS was analyzed using the Tang_2018 dataset.

### Cell culture and hypoxia treatment

4T1 and MMTV-derived primary (4T-1-P, MMTV-P) or lung metastatic (4T1-L, MMTV-L) cells were cultured in RPMI-1640 supplemented with 10% FBS (Fetal Bovine Serum). Hypoxia was induced by incubating cells at 1% O₂ for 24 h in a hypoxia chamber.

### Gene overexpression and knockdown

Mouse Cd36 cDNA plasmid (OriGene, Catalog No. MG226923) or the corresponding empty vector control (Catalog No. PS100010) was transfected into MMTV-P and 4T1-P cells to generate stable Cd36-overexpressing lines. Transfection was performed using Lipofectamine™ 2000 (Thermo Fisher Scientific, Waltham, MA, USA). For loss-of-function studies, Hif-2α (Epas1) was silenced using a SMARTpool siRNA (Dharmacon, Lafayette, CO, USA) targeting CACUUGAUGUGGAAACGUA, GGCAGUACCCAGACGGGACU, GACAAGACCAUCAGUGGCGA, and GAUAAGUACCCGGCAGCAGU. A non-targeting siRNA pool (Dharmacon) containing UGGUUUACAUGUCGACUAA, UGGUUUACAUGUUGUGUGA, UGGUUUACAUGUUUUCUGA, and UGGUUUACAUGUUUCCUA served as the negative control. Additional knockdown experiments were performed using ON-TARGETplus 2.0 SMARTpool siRNAs (Dharmacon). These included Mouse Exoc4 (20336; Catalog No. J2-051541-01, -02, -03, and -04; target sequences: UUGAAGGAAUCGAGCAUAA, AUCACUAACUCGAGGAAUA, GUACAGCGUAACAAGGAAA, and UGACUGAGUACUUGGAUAU), Mouse Sftpc (20389; Catalog No. J2-044343-01, -02, -03, and -04; target sequences: GCAUCGUUGUGUAUGACUA, CCACAGGGUCGGUAGAAAC, CAGCCUGAUUGGAAAACAA, and CCACUGGCAUCGUUGUGUA), and Mouse Cd36 (12491; Catalog No. J2-062017-01, -02, -03, and -04; target sequences: GCACAUGGCAUAAGUAUAA, GCAAUAAGUCACAGCAUAU, CGACAUGAUUAAUGGCACA, and GAUCUGAAAUCGACCUUAA). Knockdown efficiency was confirmed by qRT-PCR.

### Angiogenesis in vitro and in vivo

Tube formation assay: breast cancer cells or HUVECs (Human umbilical vein endothelial cells) were plated on reduced growth factor Matrigel-coated (Corning, cat no., 356231) plates in the presence of culture medium from control or Cd36-overexpressing cells. Tube-like structures were imaged after 4 h (4T1), 16 h (MMTV), and 16 h (HUVEC plus breast cancer cells) and quantified using ImageJ Angiogenesis Analyzer. In vivo Matrigel plug assay: 4T1-L cells were mixed with Matrigel High Concentration (#354262; Corning, NY, USA) and injected subcutaneously into BALB/c mice (*n* = 8 for each group). After 14 days, plugs were collected and stained for Cd31 and Cd36 to evaluate vascular mimicry of 4T1 in vivo.

### Cell migration assay

For the Transwell migration assay, MMTV-L and 4T1-L cells were transfected with Exoc4 siRNA or non-targeting control siRNA according to the manufacturer’s instructions. After transfection, cells were collected and resuspended in serum-free medium, and an equal number of cells were seeded into the upper chamber of 8-µm pore size Transwell inserts (Corning, NY, USA). The lower chamber was filled with culture medium supplemented with 10% FBS to serve as a chemoattractant. After incubation for 12 h, cells remaining on the upper surface of the membrane were removed using a cotton swab. Cells that had migrated to the underside of the membrane were fixed and stained with crystal violet. Migrated cells were counted in multiple random microscopic fields under a light microscope, and the average number of migrated cells was calculated.

### Transmembrane migration assay

HUVEC cells were seeded into the upper chamber of Transwell inserts (pore size, 8 µm; Corning, NY, USA) and cultured for 2 days until a confluent monolayer was formed. Cd36-overexpressing or vector control 4T1-P cells, and Cd36-knockdown or control siRNA-transfected MMTV-L and 4T1-L cells, were labeled with PKH26 dye (Sigma-Aldrich, St. Louis, MO, USA) according to the manufacturer’s instructions and then seeded onto the Transwell inserts containing HUVEC monolayer. The lower chamber was filled with culture medium containing 10% FBS as a chemoattractant. After 72 h of incubation, tumor cells that had transmigrated through the HUVEC monolayer and reached the lower surface of the insert membrane were visualized and quantified by a fluorescence microscopy.

### Quantitative real-time PCR (qRT-PCR)

RNA was extracted using TRIzol Reagent (Thermo Fisher Scientific, Waltham, MA), and cDNA was synthesized using the PrimeScript RT Reagent Kit (Takara Bio, Shiga, Japan) according to the manufacturer’s instructions. qRT-PCR was performed with SYBR Green Master Mix (Applied Biosystems, Foster City, CA) on a QuantStudio™ 3 Real-Time PCR System (Thermo Fisher Scientific, Waltham, MA). Primers were designed using the Primer3 software, and sequences are listed in Supplementary Table 1. Relative expression was normalized to *Gapdh* and calculated using the 2⁻^ΔΔCt^ method.

### Western blot, Immunohistochemistry (IHC), and Luminex analysis

Protein lysates were prepared using RIPA lysis buffer (Thermo Fisher Scientific, Waltham, MA) supplemented with protease and phosphatase inhibitors (Sigma-Aldrich, St. Louis, MO). Protein concentrations were determined using a Bicinchoninic Acid Protein Assay Kit (Millipore, Burlington, MA). Equal amounts of protein (20–50 µg) were resolved by 6–12% SDS-PAGE (sodium dodecyl sulfate-polyacrylamide gel electrophoresis) and transferred onto PVDF (polyvinylidene difluoride) membranes (Millipore, Burlington, MA). Membranes were blocked with 5% non-fat milk in TBST (Tris-buffered saline + 0.1% Tween 20), incubated overnight at 4 °C with primary antibodies against Cd36, Surfactant protein C (SP-C, encoding by *Sftpc*), Hif-2α (encoding by *Epas1*), E-cadherin, N-cadherin, vimentin, Akt and phospho-Akt and Gapdh (details in Supplementary Table 2), followed by HRP-conjugated secondary antibodies (Cell Signaling Technology, Danvers, MA) for 1 h at room temperature. Bands were detected with an Enhanced Chemiluminescent kit (Millipore, Burlington, MA) and visualized using an Azure Imaging System (Azure Biosystems, Dublin, CA).

Tumor and lung tissues were fixed in 10% formalin, paraffin-embedded, and sectioned at 5 µm. In addition, paired primary tumor and lung metastatic tissue specimens from 10 patients with triple-negative breast cancer (TNBC) were included for immunohistochemical analysis. The use of human specimens was approved by the Institutional Review Board (IRB) of Kaohsiung Medical University Hospital (KMUH) (IRB: KMUHIRB-F(I)20230169), and written informed consent was obtained from all patients. After deparaffinization and antigen retrieval, sections were incubated with primary antibodies against Cd36 (Abcam, cat. no. ab252923), Cdh5 (Abcam, cat. no. ab318152), Hif-2α (Novus Biologicals, cat. no. NB100-132), Exoc4 (Proteintech, cat. no. 11913-1-AP), and SP-C (Abcam, cat. no. ab90716), followed by HRP-conjugated secondary antibodies. Signals were detected using DAB (brown), and sections were counterstained with hematoxylin. For double IHC staining, sections were incubated with antibody combinations including Cd31 plus Cd36, Cd36 plus Cdh5, and Cd36 plus Hif-2α. Signals were visualized using distinct chromogens, including DAB and Vina Green™ (Vector Laboratories, Newark, CA, USA), allowing clear discrimination between co-localized and single-marker expression under light microscopy.

Cxcl1 and Cxcl2 protein levels in conditioned media were measured using Luminex assay kits (R&D Systems, Minneapolis, MN, U.S.A) according to the manufacturer’s instructions. Data acquisition and quantification were performed using xPONENT software (Luminex Corporation, Austin, TX, U.S.A).

### Statistical analysis

All experiments were performed at least in biological triplicate. Data were presented as mean ± SD. Comparisons between two groups were performed using Student’s t-test, as appropriate. Comparisons among more than two groups were performed using one-way ANOVA followed by Tukey’s multiple-comparison post hoc test. Survival analyses were performed using Kaplan–Meier with the log-rank test. A *p*-value < 0.05 was considered statistically significant.

## Results

### Identification of metastasis-associated cancer cell phenotypes in TNBC

**Fig. 1:**
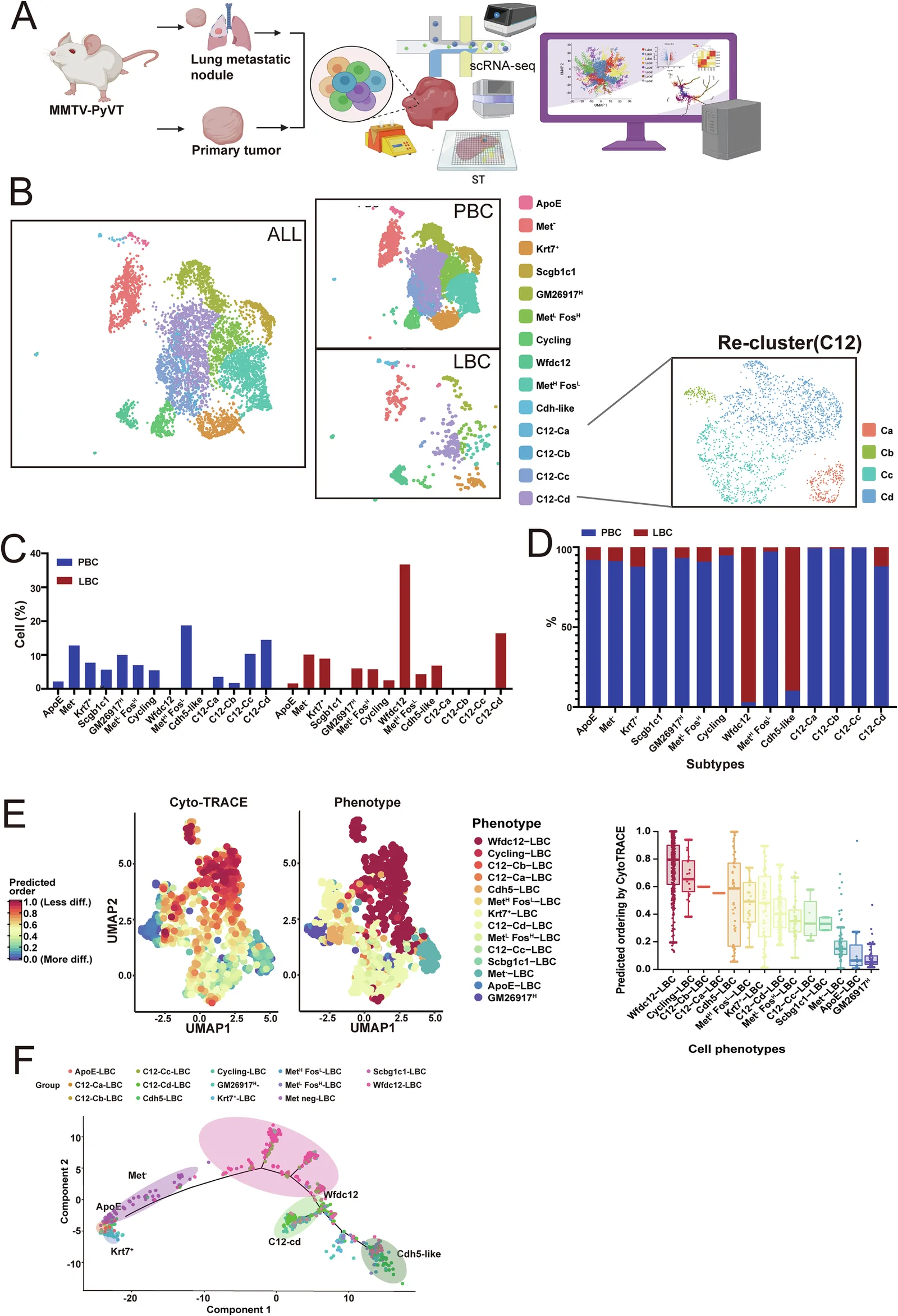
Single-cell transcriptomic profiling of primary and metastatic TNBC. **A** Schematic overview of the study design. Paired specimens from MMTV-derived primary mammary tumors and matched lung metastases were collected for single-cell RNA sequencing and spatial transcriptomics. **B** UMAP identified 14 cancer subtypes of breast cancer in MMTV-PyVT model. **C** Percentages of the various cancer subclusters in primary and lung metastatic sites. **D** The proportions of major subtypes were shown for primary tumors versus lung metastases. **E** CytoTRACE analysis of differentiation states of 14 cancer subtypes in lung metastatic sites. **F** Trajectory analysis positioned Wfdc12, Cdh5-like, and C12-Cd clusters at late stages of metastatic progression. PBC, primary breast cancer; LBC, lung metastatic breast cancer.

To investigate the cellular transition from primary to metastatic TNBC, we performed scRNA sequencing and spatial transcriptomics (ST) on paired MMTV-derived mammary tumors and matched lung metastases (Fig. 1A). *Epcam*⁺ epithelial cells were selected, with AT1 and AT2 cells excluded. Unsupervised clustering identified 14 cancer subtypes, including ApoE, Met⁻, Krt7^+^, Scgb1c1, GM26917^H^, Met^L^ Fos^H^, cycling (proliferative), Wfdc12, Met^H^ Fos^L^, Cdh5-like, and four C12 subclusters (C12-Ca, C12-Cb, C12-Cc, C12-Cd) (Fig. 1B). In primary tumors, Met⁻, Met^H^ Fos^L^, and C12-series cells predominated, whereas Wfdc12 cells, and to a lesser extent C12-Cd cells, were enriched in lung metastases (Fig. 1C). Analysis of cell distribution across tissue origins further showed that C12-Cd cells were present in both primary tumors and lung metastases, consistent with metastatic potential, while Wfdc12 and Cdh5-like cells were largely restricted to lung metastases, indicating a lung-specific phenotype (Fig. 1D). CytoTRACE analysis revealed that Wfdc12 and cycling clusters exhibited lower differentiation states, whereas ApoE and GM26917^H^ clusters in lung lesions showed relatively higher differentiation levels (Fig. 1E). Trajectory analysis further positioned Wfdc12, Cdh5-like, and C12-Cd clusters toward the terminal branch of the inferred metastatic progression trajectory (Fig. 1F). Together, these findings suggested that Wfdc12, Cdh5-like, and C12-Cd clusters are associated with metastatic states in the lung.

### Upregulation of Exoc4 in the BC derived lung metastasis and correlated with worse prognosis

**Fig. 2:**
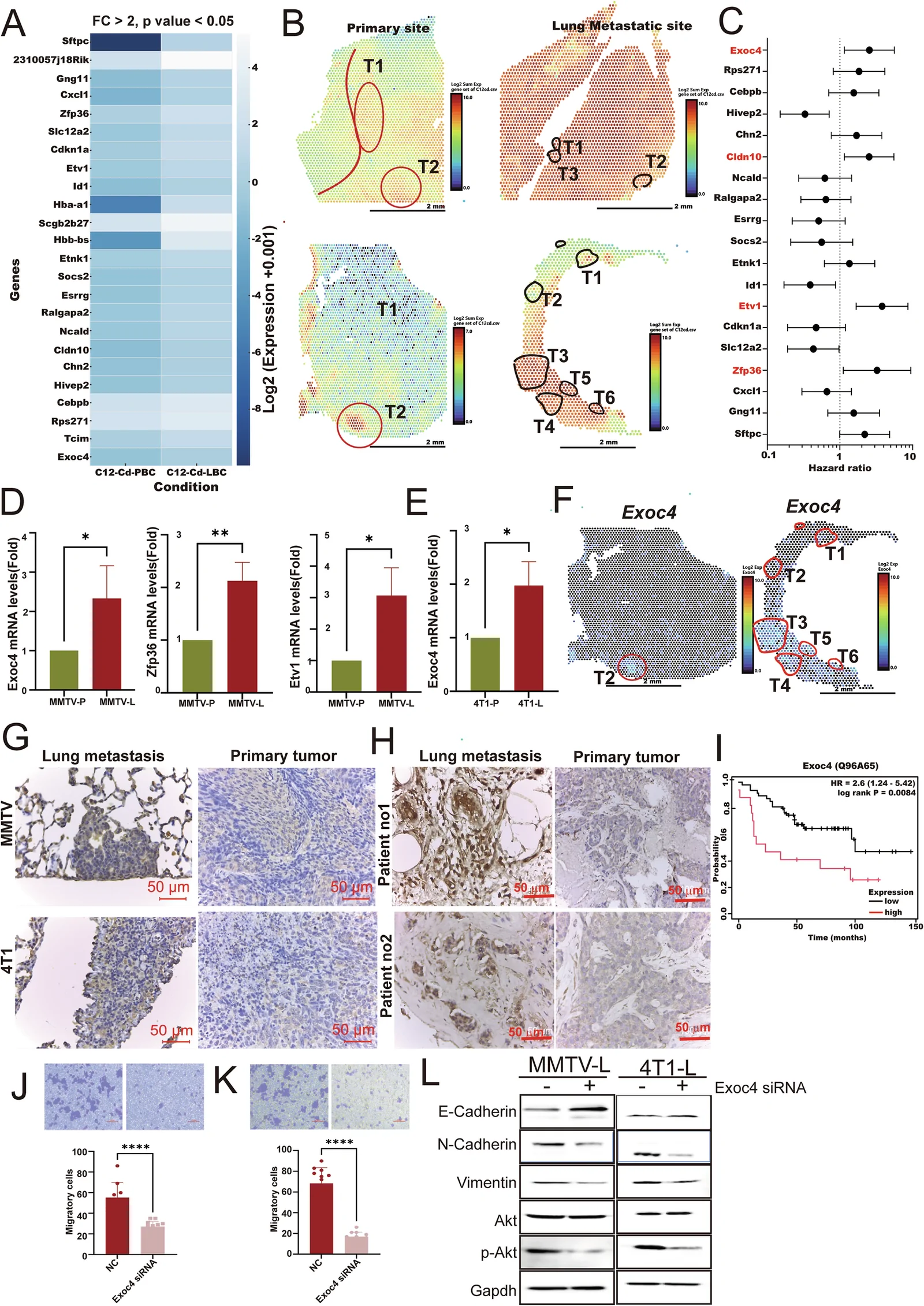
Identification of C12-Cd subcluster–associated DEGs and validation of Exoc4 as a metastasis-related gene in TNBC. **A** Heatmap of the differentially expressed genes of C12-Cd subcluster between primary (PBC) and lung metastatic lesions (LBC) (fold change > 2, *p* < 0.05). **B** Spatial transcriptomics revealed restricted expression of the 24-gene set in a subregion of the primary tumor and lung metastatic lesions. **C** Four genes—*Exoc4*, *Cldn10*, *Etv1*, and *Zfp36*—were associated with worse overall survival (OS) in TNBC patients. Expression of these four genes was validated in MMTV (**D**) and 4T1 mouse models (**E**). **F** Elevated *Exoc4* expression was enriched in the in tumor of lung metastatic sites. IHC confirmed increased expression of Exoc4 protein in metastatic lung nodules in mouse model (**G**) and paired human primary breast cancer and lung metastasis specimens (**H**). **I** High Exco4 protein levels correlated with poorer OS in TNBC patients. Transwell migration assay showing that Exoc4 knockdown markedly reduced the migratory ability of MMTV-L (**J**) and 4T1-L (**K**) cells compared with control cells. **L** Immunoblot analysis showing that Exoc4 knockdown suppressed EMT and Akt activation in MMTV-L and 4T1-L cells, as indicated by reduced phosphorylated Akt levels. Bars indicated mean ± SD. For two-group comparisons, unpaired Student’s *t*-test was used. Significance: *p* < 0.05 (*), *p* < 0.01 (**), *p* < 0.001 (***), *p* < 0.0001 (****).

Because the C12-Cd sub-cluster exhibited metastatic potential to the lung, we next analyzed the DEGs of C12-Cd clusters between the primary site and metastatic lesions. We focused on upregulated genes because they are more likely to reflect activated biological programs associated with metastatic progression and are generally more amenable to functional interpretation. As shown in Fig. 2A, 24 genes (fold change > 2, *p* < 0.05) were significantly upregulated in the lung metastatic site. Spatial transcriptomics further confirmed that this 24-gene set was highly expressed in a restricted region of the primary tumor, and became broadly elevated in lesions of breast cancer lung metastasis (Fig. 2B and Supplementary Fig. 2A). Using the TCGA cohort, we investigated the association between these 24 genes and overall survival (OS) in TNBC patients. Four genes—*Exoc4*, *Cldn10*, *Etv1*, and *Zfp36*—were significantly correlated with poorer OS in the patients with TNBC (Fig. 2C, *p* < 0.05). *Exoc4*, *Etv1*, and *Zfp36* were significantly upregulated in MMTV-L cells relative to MMTV-P cells (Fig. 2D). Notably, *Exoc4* upregulation was further confirmed in 4T1-L cells compared with 4T1-P cells, whereas *Cldn10* showed no statistically significant difference in either the MMTV-PyMT or 4T1 model. (Fig. 2E). Elevated *Exoc*4 expression was particularly enriched in the region expressing C12-Cd gene set at both the primary (T2) and metastatic tumor nodules (T3–T5) (Fig. 2F and Supplementary Fig. 2B). IHC further validated that Exoc4 expression was markedly increased in metastatic lung nodules of both the MMTV transgenic and the 4T1 orthotopic models compared to their corresponding primary tumors (*n* = 5, Fig. 2G and Supplementary Fig. 2C, D). Consistently, paired human primary breast cancer and lung metastasis specimens showed the same pattern (Fig. 2H). High Exoc4 levels were also associated with worse OS in TNBC patients (Fig. 2I). Knockdown of Exoc4 in both MMTV-L and 4T1-L cells decreased migratory ability, mesenchymal marker expression (N-cadherin and Viemtin), and Akt activation (Fig. 2J–L and Supplementary Fig. 2E), suggesting that Exoc4 contributes to TNBC lung metastasis through the promotion of migration and EMT. Together, these findings identified Exoc4 as a critical metastasis-associated gene within the C12-Cd subcluster, linking its upregulation to lung metastasis and poor clinical outcomes in TNBC.

### Cdh5-like subcluster defined a vascular-associated population enriched in lung metastasis

**Fig. 3:**
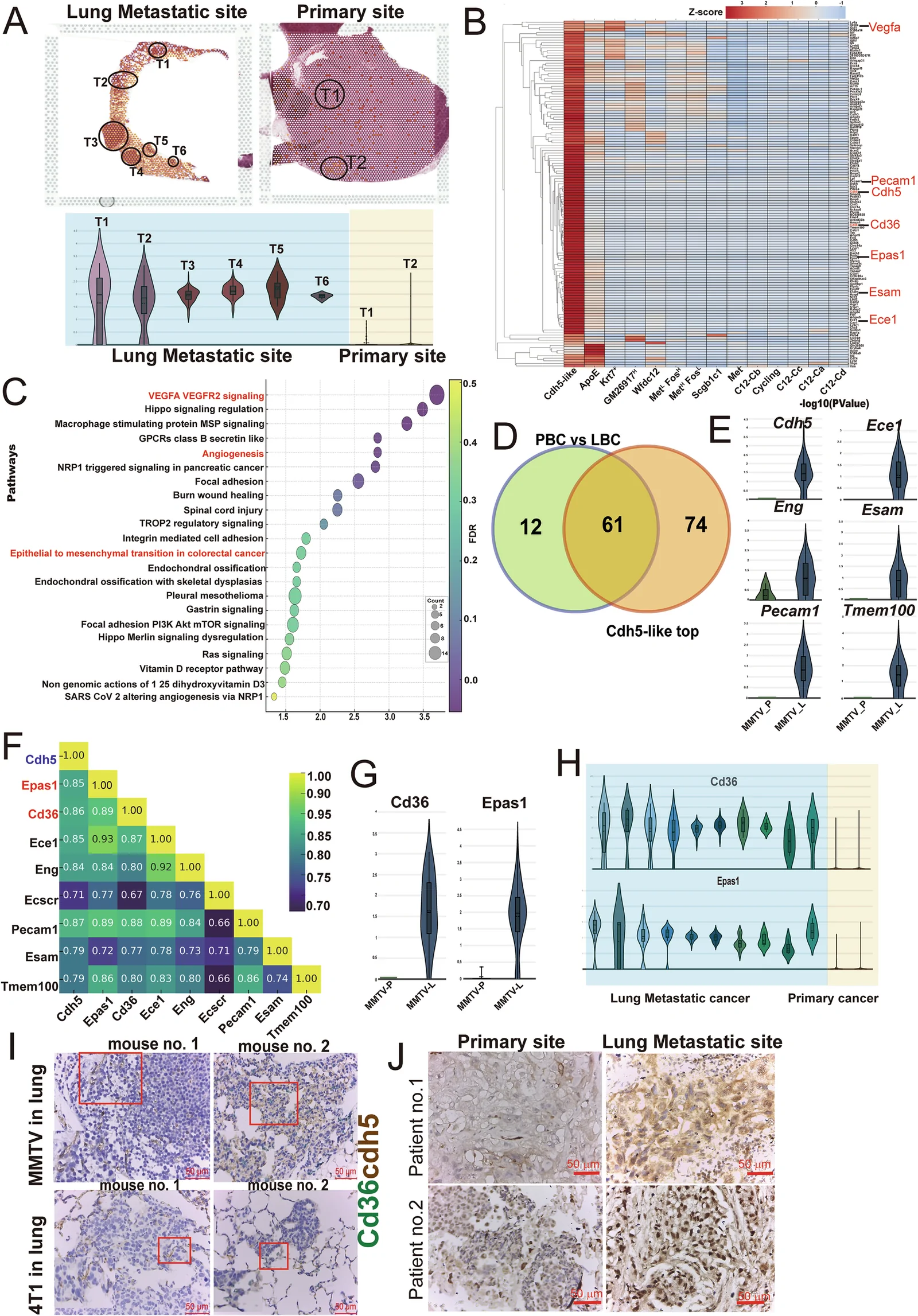
Characterization of the Cdh5-like subcluster and its vascular-associated gene program. **A** *Cdh5* expression in lung metastatic sites and primary BC. **B** Heatmap showing differentially expressed genes (DEGs) of the Cdh5-like subcluster compared to other subclusters, identifying 135 upregulated genes. **C** KEGG pathway enrichment of the upregulated genes in Cdh5-like subclusters. **D** Intersection analysis identified 61 overlapping DEGs between primary and lung metastatic sites. **E** Vascular physiology–related genes (*Cdh5, Ece1*, *Eng*, *Esam*, *Pecam1*, and *Tmem100*) were significantly upregulated in Cdh5-like subcluster of lung metastatic cancer compared to primary tumor. **F** Correlation matrix showing strong correlation among vascular physiology genes. **G** *Cd36* and *Epas1* were also upregulated in in Cdh5-like subcluster of lung metastatic cancer than in primary tumor. **H** Spatial transcriptomics revealed upregulation of *Cd36* and *Epas1* in lung metastatic lesions. **I** IHC validated co-expression of Cd36 and Cdh5 in metastatic lung nodules of both 4T1 and MMTV mouse models. **J** IHC confirmed increased expression of CD36 protein in metastatic lung nodules in paired human primary breast cancer and lung metastasis specimens.

Because the Cdh5-like cluster represented a distinct subpopulation in TNBC, we next profiled its transcriptional features relative to other subclusters. ST corroborated that metastatic lung nodules exhibited high *Cdh5* expression, whereas primary tumors showed low *Cdh5* expression (Fig. 3A). Differential expression gene analysis identified 135 genes significantly upregulated in the Cdh5-like subcluster compared to the primary sites (Fig. 3B). Pathway enrichment pointed to strong associations with “VEGFA–VEGFR2 signaling”, “Angiogenesis”, and “Epithelial to mesenchymal transition in colorectal cancer” (Fig. 3C). Intersecting the top-ranked DEGs from Cdh5-like subclusters at the primary and lung metastatic sites yielded 61 shared genes (Fig. 3D). These included vascular physiology–related genes—*Cdh5*, *Ece1*, *Eng*, *Esam*, *Pecam1*, and *Tmem100*—all of which were expressed at higher levels in the lung metastasis Cdh5-like subcluster than in primary tumors (Fig. 3E). Notably, expression of these genes within cancer cells of the Cdh5-like subcluster was strongly correlated (*R* > 0.67, *p* < 0.01) (Fig. 3F). Beyond classical vascular markers, *Cd36* and *Epas1* (Hif-2α), also displayed strong correlations and were consistently upregulated in the Cdh5-like subcluster of lung metastases relative to primary tumors (Fig. 3G). ST further supported high *Cd36* and *Epas1* expression in metastatic nodules and low Cdh5 expression at the primary site (Fig. 3H). Finally, IHC confirmed co-expression of Cd36 and Cdh5 in metastatic lung nodules in both the MMTV transgenic and 4T1 orthotopic models (*n* = 5, Fig. 3I). Moreover, in paired human TNBC specimens, CD36 expression was markedly higher in lung metastatic nodules than in the matched primary tumor tissues (Fig. 3J). These data supported Cd36 might play an important role in TNBC lung metastasis.

### Hif-2α mediated hypoxia-driven Cd36 and angiogenic gene expression

**Fig. 4:**
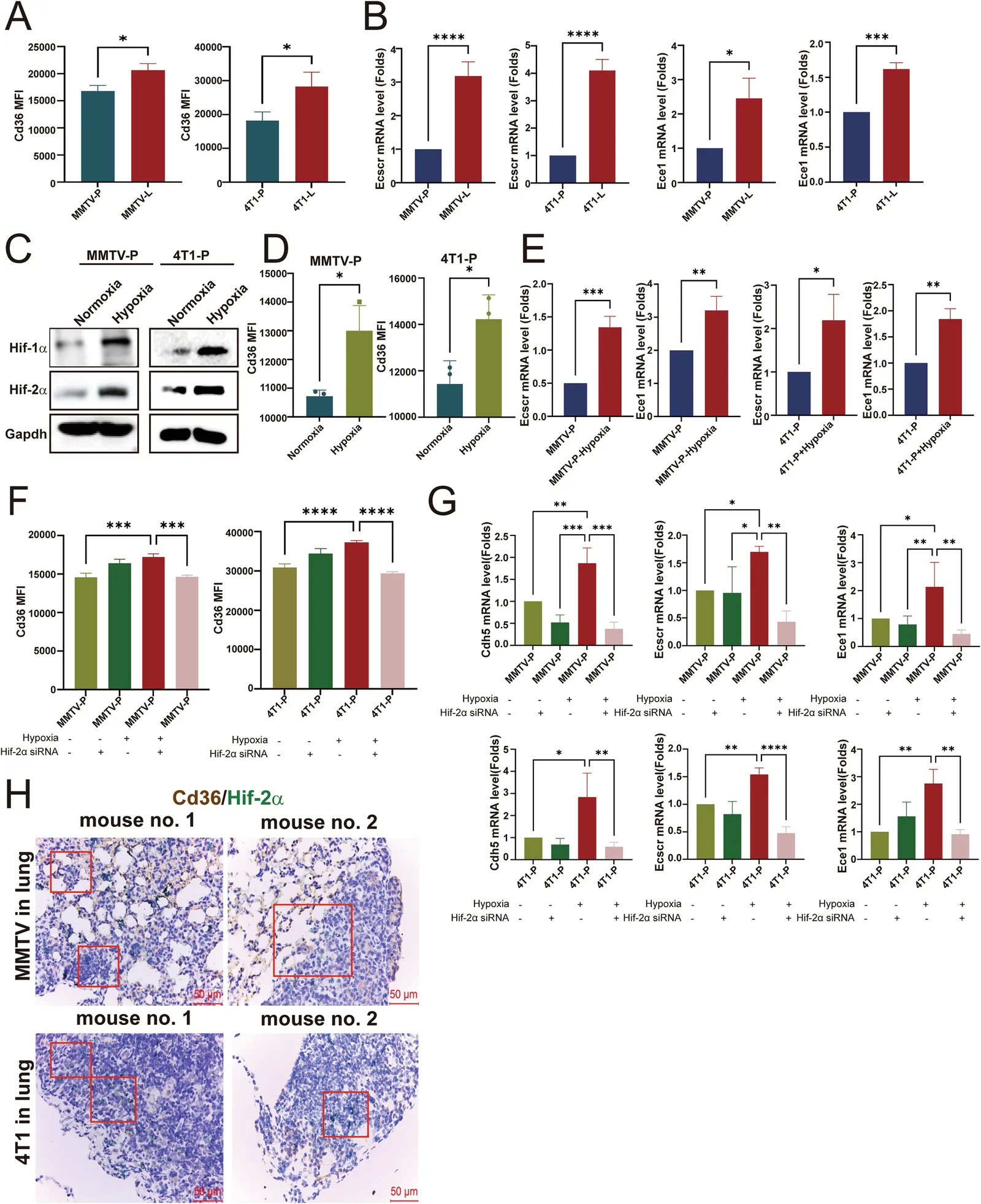
Hypoxia promoted Cd36 expression and induced vascular-associated genes in metastatic breast cancer cells. **A** Flow cytometry analysis showing upregulation of Cd36 in metastatic-derived cells (MMTV-L and 4T1-L) compared to their primary counterparts (MMTV-P and 4T1-P). MFI mean fluorescence intensity. **B** Increased expression of vascular physiology–related genes (*Ecscr* and *Ece1*) in metastatic-derived cells. **C** Hypoxic culture conditions (1% O₂) induced Hif-2α expression. **D** Hypoxia increased *Cd36* expression in MMTV-P and 4T1-P cells. **E** Hypoxia also enhanced the expression of vascular-associated genes (*Ecscr* and *Ece1*) in MMTV-P and 4T1-P cells. Knockdown of HIf-2α by siRNA prevented hypoxia-induced upregulation of Cd36 protein (**F**), and *cdh5*, *Ece1* and *Ecscr mRNA* expression (**G**). **H** IHC confirmed co-expression of Cd36 and Hif-2α in metastatic lung nodules of both MMTV-P transgenic and 4T1-P orthotopic models. Bars indicated mean ± SD. For two-group comparisons, unpaired Student’s t-test was used. For comparisons involving more than two groups, one-way ANOVA with Tukey’s post hoc test was used. Significance: *p* < 0.05 (*), *p* < 0.01 (**), *p* < 0.001 (***), *p* < 0.0001 (****).

To further verify the association between the Cdh5-like subcluster and hypoxia, we employed an ex vivo model in which flow cytometric analysis showed significantly increased Cd36 expression at protein level in MMTV-L and 4T1-L cells compared to their primary counterparts (MMTV-P and 4T1-P) (Fig. 4A). Similarly, vascular physiology–related genes, such as *Ecscr* and *Ece1* were also elevated in metastatic-derived cells (Fig. 4B). When MMTV-P and 4T1-P cells were cultured under a hypoxic condition (1% O₂), we observed induction of Hif-2α (Fig. 4C), accompanied by increased expression of Cd36 (Fig. 4D). In addition, hypoxia also enhanced the expression of vascular-related genes, including *Cdh5*, *Ecscr* and *Ece1* in both MMTV-P and 4T1-P cells (Fig. 4E). Importantly, siRNA-mediated Hif-2α knockdown abolished the hypoxia-induced upregulation of Cd36 at the protein level, as well as *Ecscr* and *Ece1* at the mRNA level (Fig. 4F, G and Supplementary Fig. 3A, B). IHC confirmed the co-expression of Cd36 and Hif-2α in metastatic lung nodules of both 4T1 orthotopic and MMTV transgenic models (Fig. 4H), indicating that hypoxia promotes Cd36 expression and enhances the expression of vascular-associated molecules.

### Cd36 as a key promoter of angiogenesis and metastatic progression in breast cancer

**Fig. 5:**
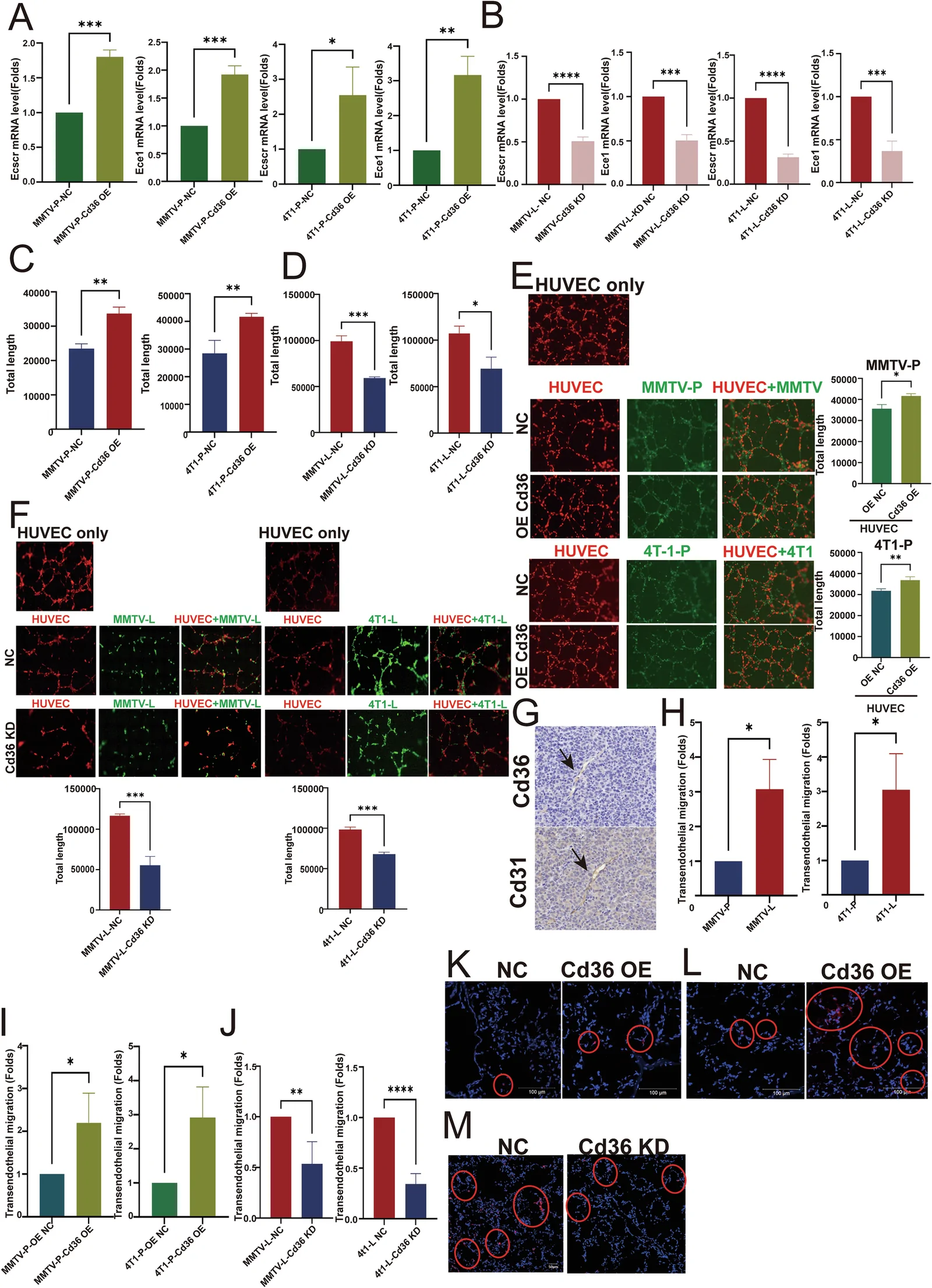
Cd36 promoted angiogenesis, vascular mimicry, and metastatic colonization in breast cancer. **A** Overexpression of Cd36 in MMTV-P and 4T1-P cells increased the expression of vascular markers, *Ecscr* and *Ece1*. **B** qRT-PCR analysis showing the knockdown of Cd36 decreased the expression of *Ece1* and *Ecscr* in MMTV-L and 4T1-L cells. **C** Cd36 overexpression enhanced the angiogenic ability of MMTV-P and 4T1-P cells. **D** Cd36 knockdown decreased the angiogenic ability of MMTV-L and 4T1-L cells. **E** Representative images and quantification of HUVEC tube formation induced by Cd36-overexpressing MMTV-P and 4T1-P cells. **F** Representative images and quantification of HUVEC tube formation induced by Cd36-knockdown MMTV-L and 4T1-L cells. **G** In vivo Matrigel plug assay showed that Cd36⁺ 4T1 cells, together with CD31⁺ endothelial cells, formed vessel-like structures in the Matrigel plug of mice. **H** MMTV-L and 4T1-L cells displayed greater ability of transendothelial migration. **I** Cd36-overexpressing MMTV-P and 4T1-P cells showed enhanced transendothelial migration ability. **J** Cd36-knockdown MMTV-L and 4T1-L cells showed decreased transendothelial migration ability. Cd36-overexpressing 4T1-P cells exhibited improved lung colonization at 24 h (**K**) and 7 days (**L**) after tail-vein injection in mice. **M** Cd36-knockdown 4T1-L cells exhibited improved lung colonization at 24 h. Bars indicated mean ± SD. For two-group comparisons, unpaired Student’s t-test was used. Significance: *p* < 0.05 (*), *p* < 0.01 (**), *p* < 0.001 (***). NC control plasmid. NC normal control (scramble siRNA for KD or empty cDNA plasmid for OE), KD knockdown, OE over expression. The circles indicate areas of tumor cell localization in the lungs after tail vein injection.

To further investigate whether Cd36 regulates vascular marker expression and angiogenic activity, we performed both Cd36 overexpression and knockdown experiments in MMTV and 4T1 cells. As shown in Fig. 5A, Cd36 overexpression significantly increased the expression of *Ecscr* and *Ece1* in both MMTV-P and 4T1-P cells, whereas Cd36 knockdown reduced their expression in MMTV-L and 4T1-L cells (Fig. 5A, B). In parallel, Cd36 overexpression enhanced the angiogenic capacity of MMTV-P and 4T1-P cells, whereas Cd36 knockdown suppressed the angiogenic ability of MMTV-L and 4T1-L cells (Fig. 5C, D and Supplementary Fig. 4A, B). Consistently, Cd36 overexpression in MMTV-P and 4T1-P cells promoted HUVEC tube formation, whereas Cd36 knockdown attenuated the pro-angiogenic effects of MMTV-L and 4T1-L cells on HUVECs (Fig. 5E, F), supporting the role of Cd36 in promoting vascular mimicry in breast cancer. An in vivo angiogenesis assay further supported that Cd36-positive 4T1 cells, together with Cd31⁺ vascular cells, formed vessel-like structures in the mouse subcutaneous Matrixgel plug (Fig. 5G). Compared to breast cancer cells isolated from the primary site, lung-derived MMTV-L and 4T1-L cells exhibited higher transendothelial migration capacity (Fig. 5H). Likewise, Cd36 overexpression enhanced the transendothelial migration ability of MMTV-P and 4T1-P cells (Fig. 5I), whereas Cd36 knockdown suppressed transendothelial migration in both MMTV-L and 4T1-L cells (Fig. 5J). More importantly, 4T1-P cells overexpressing Cd36 exhibited enhanced lung colonization in mice at both 24 h and 7 days after tail-vein injection (Fig. 5K, L), whereas Cd36 knockdown attenuated lung colonization of 4T1-L (Fig. 5M). These findings demonstrated that Cd36 promoted vascular mimicry and metastatic colonization during lung metastasis in breast cancer.

### The Wfdc12 subcluster secreted Cxcr2 ligands to promote TNBC lung metastasis

**Fig. 6:**
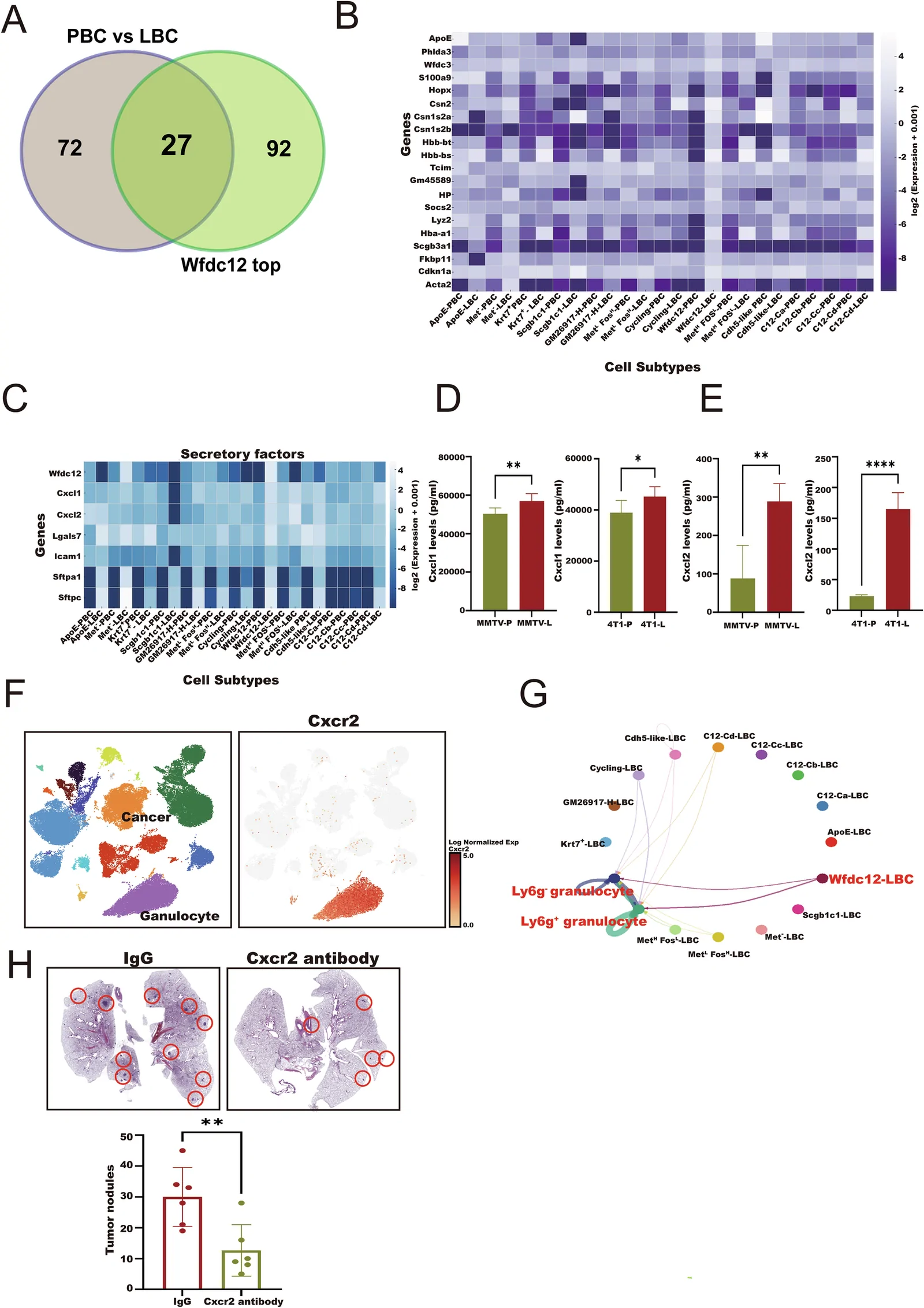
Wfdc12 cluster promoted lung metastasis through Cxcl1/2–Cxcr2 signaling. **A** Venn diagram showing the intersection of top-ranked DEGs (FC > 2, *p* value < 0.05) in Wfdc12 clusters between primary tumors and lung metastases. **B** Heatmap of 27 shared genes, many of which encode secretory proteins. **C** Representative examples of secretory genes enriched in Wfdc12 clusters. Luminex analysis validation showing elevated Cxcl1 (**D**) and Cxcl2 (**E**) protein levels in lung-derived MMTV-L and 4T1-L cells compared to primary-site MMTV-P and 4T1-P cells. **F** Granulocytes were identified as the predominant Cxcr2⁺ population in the lung metastatic microenvironment. **G** Cell–cell interaction analysis revealed Cxcl-mediated crosstalk between Wfdc12 subcluster of TNBC cells and Ly6g⁺/Ly6g− granulocytes. **H** Neutralization of Cxcr2 markedly reduced TNBC lung metastasis in the 4T1 model. Bars indicate mean ± SD. For two-group comparisons, unpaired Student’s t-test was used. *p* < 0.05 (*), *p* < 0.01 (**), *p* < 0.0001 (****).

Another unique lung metastatic subcluster identified was the Wfdc12 cluster. By intersecting the top-ranked DEGs (FC > 2, *p*-value < 0.05) between primary and lung metastatic sites (FC > 2, *p*-value < 0.05), we identified 27 shared genes (Fig. 6A, B and Supplementary Table 3), many of which encoded secretory proteins such as *Cxcl1*, *Cxcl2*, *Icam1*, *Lgals7*, *Sftpa1*, and *Sftpc* (Fig. 6C). Our data further confirmed that MMTV-L and 4T1-L cells produced significantly higher levels of Cxcl1 and Cxcl2 secretion compared to their primary counterparts (Fig. 6D, E). Within the lung microenvironment, granulocytes were identified as the predominant Cxcr2⁺ population (Fig. 6F). CellChat interaction analysis further revealed that Wfdc12 subcluster TNBC cells engaged in Cxcl-mediated crosstalk with both Ly6g⁺ and Ly6g⁻ granulocytes (Fig. 6G). Functionally, treatment with a Cxcr2 neutralizing antibody markedly reduced lung metastasis in the MMTV transgenic model (Fig. 6H). Together, these findings defined the Wfdc12 subcluster as a metastasis-associated TNBC population that promoted lung colonization through secretion of Cxcr2 ligands and paracrine activation of granulocytes.

### Sftpc (Surfactant protein C, SP-C) was defined as a lung metastasis–specific marker in TNBC

**Fig. 7:**
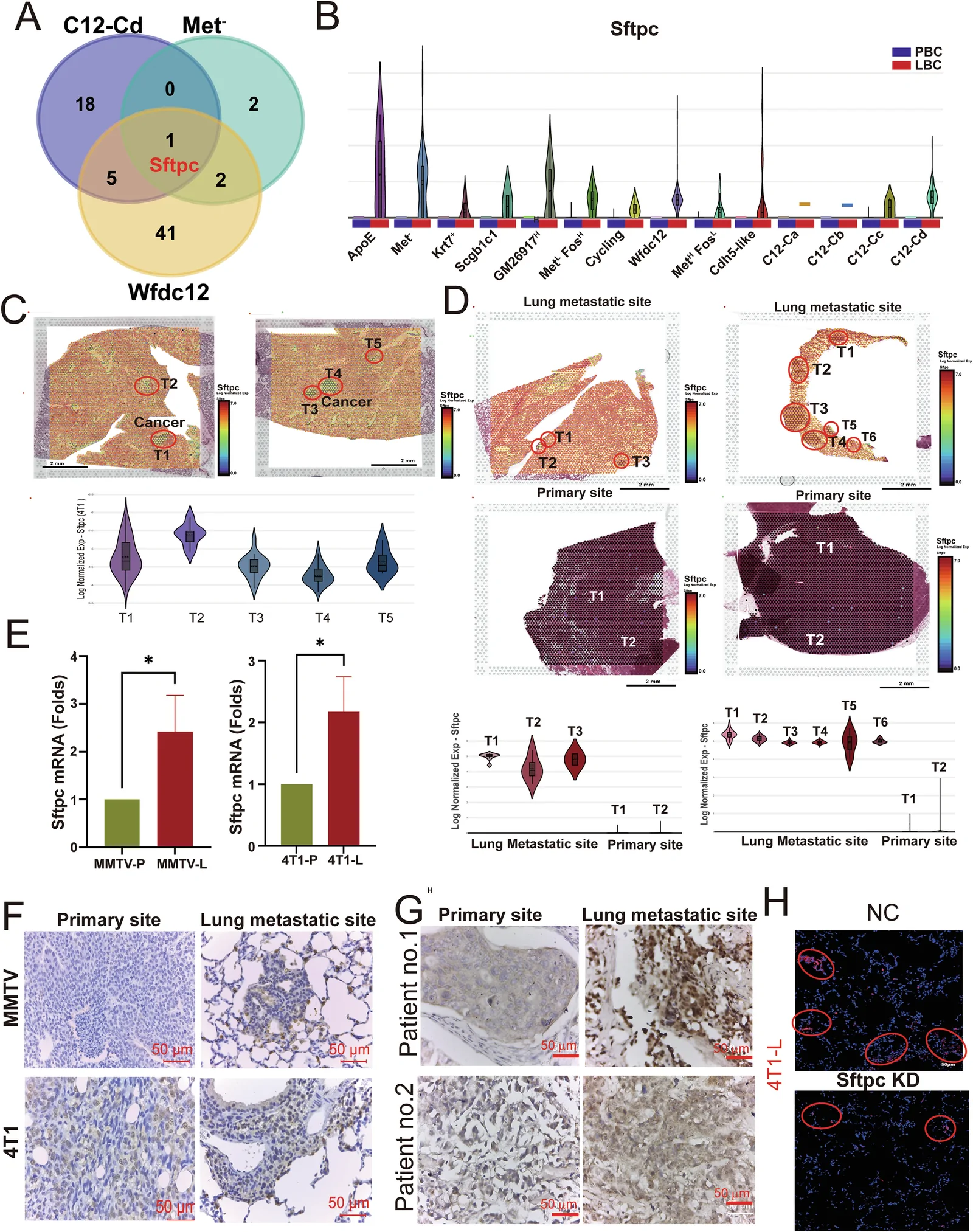
SP-C was a common gene expressed explicitly in lung metastatic TNBC clusters. **A** Intersection of the top DEGs from the three largest metastatic subclusters in lungs of mice. **B** *Sftpc* was broadly expressed across all lung metastatic subclusters but was nearly absent in subclusters from primary-site TNBC. Spatial transcriptomics revealed *Sftpc* expression in lung metastatic nodules of both the 4T1 mouse model (**C**) and the MMTV model (**D**), but not in primary tumor nodules. **E** qRT-PCR confirmed higher *Sftpc* mRNA. **F** IHC staining demonstrated SP-C expression in lung metastatic TNBC lesions in both the MMTV transgenic and 4T1 orthotopic mouse models. **G** Representative IHC staining showing high SP-C expression in human TNBC lung metastatic specimens. **H** siRNA-mediated knockdown of *Sftpc* significantly suppressed lung colonization of 4T1-L cells after 24 h injection. Bars indicated mean ± SD. For two-group comparisons, unpaired Student’s t-test was used. *p* < 0.05 was considered significant. Significance: *p* < 0.05 (*). The circles indicated areas of tumor cell localization in the lungs after tail vein injection.

Finally, to explore whether common genes are shared across different subclusters of lung metastatic tumors, we intersected the top DEGs of the three main subclusters of lung metastatic site. Strikingly, only one common gene, *Sftpc*, was identified (Fig. 7A). *Sftpc* was broadly expressed across all lung metastatic subclusters, but was almost absent in the subclusters of primary-site TNBC (Fig. 7B). ST further revealed strong *Sftpc* expression in lung metastatic nodules of the 4T1 mouse model (Fig. 7C) and in the MMTV model, but not in primary tumor nodules (Fig. 7D). qRT-PCR confirmed that *Sftpc* mRNA (Fig. 7E) levels were elevated in MMTV-L and 4T1-L cells compared to their primary-sites, MMTV-P and 4T1-P, respectively. Consistently, IHC demonstrated SP-C expression in lung metastatic TNBC lesions in both the MMTV transgenic and 4T1 orthotopic mouse models (Fig. 7F and Supplementary Fig. 5A, B). In addition, strong SP-C expression was also observed in human TNBC specimens with lung metastases (Fig. 7G). More importantly, siRNA-mediated knockdown of Sftpc in 4T1-L cells markedly suppressed their lung colonization ability (Fig. 7H and Supplementary Fig. 5C). Together, these findings highlight Sftpc (SP-C) as a lung metastasis-specific marker of TNBC.

## Discussion

Lung metastasis remains a major cause of mortality in patients with TNBC. Yet, the cellular programs that enable tumor cells to colonize in the lung are not fully understood. In this study, we integrated single-cell RNA sequencing and spatial transcriptomics to delineate the cellular heterogeneity of TNBC lung metastasis. We identified three metastasis-associated subclusters—C12-Cd, Cdh5-like, and Wfdc12—each enriched in metastatic lesions and displaying distinct functional programs. The C12-Cd subcluster was linked to Exoc4 upregulation and poor prognosis, while the Cdh5-like cluster exhibited a hypoxia-driven vascular phenotype involving Cd36 upregulation. Notably, the Wfdc12 cluster was characterized by secretion of Cxcr2 ligands, and blockade of Cxcr2 with a neutralizing antibody significantly reduced lung metastasis, underscoring its therapeutic relevance. In addition, cross-subcluster analysis revealed Sftpc as a lung-specific marker of metastatic TNBC, suggesting organ-adaptive transcriptional reprogramming within the pulmonary niche. Collectively, these findings provide a framework for understanding the metastatic evolution of TNBC and highlight potential molecular vulnerabilities for targeted intervention.

In our study, we identified the C12-Cd subcluster, which was present in both the primary tumor and lung metastatic sites but exhibited selective upregulation of a distinct gene set. Spatial transcriptomics revealed that this gene program was detectable within restricted regions of the primary tumor, yet became markedly amplified within the lung metastatic niche, underscoring its potential functional significance in driving TNBC lung metastasis. Notably, high *Exoc4* expression was associated with worse overall survival in TNBC patients, highlighting its potential clinical relevance. Exoc4, also known as Sec8, is a core component of the exocyst complex, which plays a crucial role in tethering secretory vesicles to the plasma membrane before SNARE-mediated fusion [15, 16]. Beyond its canonical role in vesicle trafficking, Exoc4 has recently been implicated in anti-DNA viral immunity through activation of stimulator of interferon response cGMP interactor [17]. Dysregulated Exoc4 expression has also been reported in several malignancies; for instance, it is highly expressed in gastric cancer and correlates with recurrence and poor prognosis [18]. Moreover, both Exoc4 and Exoc7 have been implicated in resistance to neutrophil-mediated antibody-dependent cellular cytotoxicity [19]. Collectively, these findings suggested that Exoc4 overexpression within the C12-Cd subcluster of lung-metastatic TNBC further supports a pivotal role in metastatic adaptation, highlighting its potential as a prognostic biomarker and therapeutic target.

Rather than relying on endothelial cells, certain aggressive cancers acquire the ability to generate vessel-like networks independently, a process known as vasculogenic mimicry (VM) [20, 21]. VM has been implicated in tumor dissemination and resistance to anti-angiogenic therapies, and its loosely organized vascular structures may further facilitate tumor cell extravasation and colonization at metastatic sites [20]. Hypoxia is a well-recognized driver of VM [21] and recent studies have demonstrated that hypoxia-induced VM is closely linked to alterations in fatty acid metabolism [22, 23]. Among the key regulators, Cd36 functions as a critical mediator of hypoxia-driven lipid metabolic reprogramming via Hif-1α or Hif-2α signaling [24, 25] and has previously been implicated in VM formation in melanoma [26]. In our study, we identified a TNBC subcluster within the lung metastatic niche that displayed high expression of Hif-2α, a vessel-like phenotype, and Cd36. Functional assays demonstrated that hypoxia induced Cd36 expression in lung-metastatic TNBC cells, while Cd36 overexpression enhanced vascular gene expression and promoted VM capacity in vitro. In vivo, Cd36⁺ TNBC cells cooperated with CD31⁺ endothelial cells to form vascular-like structures, and Cd36 overexpression further facilitated tumor cell adhesion and colonization in the lung in a mouse model. Together, these results establish Cd36 as a hypoxia-induced driver of vasculogenic mimicry and metastatic colonization in TNBC, highlighting a critical hypoxia (Hif-2α)–Cd36–VM axis that fuels lung metastasis.

Surfactant Protein C (SP-C), encoded by the Sftpc gene, is a critical component of alveolar homeostasis that enhances the interfacial adsorption of lung surfactant lipid films at the air–liquid interface [27]. SP-C is uniquely expressed in the lung, predominantly produced by type II pneumocytes. Pathogenic Sftpc (SP-C) mutations have been linked to interstitial lung disease and pulmonary fibrosis [28, 29]. More recently, reduced expression or mutations of Sftpc have also been implicated in the development of lung cancer [30, 31]. Beyond lung cancer, members of the SFTP family have been reported to play diverse roles in other malignancies. For example, Sftpd (SP-D) has been predicted to promote lung metastasis in colorectal cancer [32]. while elevated SP-D expression correlated with shorter overall survival in luminal-A grade 1–2 breast cancers [33]. Similarly, high Sftpc expression in melanoma was associated with improved survival outcomes, including overall and disease-specific survival [34]. In our study, scRNA-seq and spatial transcriptomics analyses revealed that breast cancer cells metastasized to the lung consistently expressed Sftpc, regardless of their subclonal identity. This was further validated by IHC staining, confirming SP-C expression in metastatic 4T1 and MMTV mouse models cells within lung nodules. These findings aligned with accumulating evidence that metastatic tumor cells frequently acquire expression of organ-specific genes after colonization—for example, osteoblast- or osteoclast-related genes in bone metastases [35] and neuronal-specific genes in brain metastases, which may even endow cancer cells with neuronal-like features such as axon-like morphology, environmental cue sensing, secretory signaling, and electrical excitability [36]. Such phenomena highlight the pivotal role of organ-adaptive transcriptional reprogramming in metastatic progression. Our results suggested that the expression of Sftpc in lung-metastatic breast cancer cells may represent an adaptive mechanism within the pulmonary microenvironment, and this novel observation warrants further mechanistic investigation.

Several limitations should be acknowledged in this study. First, cancer cells can undergo different degrees of EMT during metastatic progression, and partial EMT has been suggested to confer higher invasive and metastatic potential than full EMT (EpCAM negative expression) in certain contexts [37, 38]. Recent studies have also shown that increased mesenchymal markers vimentin expression, even without loss of epithelial features such as E-cadherin and EpCAM, can enhance cancer cell invasion [39]. In our study, EpCAM-positive cells were used as the criterion for cancer cell selection; therefore, cancer cells that had undergone full EMT and lost EpCAM expression may have been excluded, potentially underestimating their contribution to lung metastasis. Second, *EXOC4* is known to be involved in cellular secretion [15, 16]. Our findings showed that *EXOC4* expression was associated with lung metastasis and poorer patient prognosis, and genetic inhibition further linked EXOC4 to EMT regulation and AKT signaling in breast cancer cells. However, whether EXOC4 upregulation promotes lung metastasis by altering tumor cell secretion and mediating autocrine or paracrine effects, or through direct intracellular regulatory mechanisms, remains unclear and requires further investigation. Third, *SFTPC* is a lung epithelial cell lineage-specific gene that plays an essential role in alveolar function [40]. Although previous studies have indicated that metastatic tumor cells may acquire organ-mimicry features to improve adaptation to a new metastatic environment [41, 42]. Whether *SFTPC* plays a similar role in TNBC lung metastasis remains to be determined. Further studies are needed to clarify whether *SFTPC* contributes to lung colonization and metastatic niche adaptation in triple-negative breast cancer.

In summary, our work provides a comprehensive cellular and molecular atlas of TNBC lung metastasis linking hypoxia, vesicle trafficking, vasculogenic mimicry, and secretory programs to the progression of metastasis. By integrating single-cell, spatial, and functional validation approaches, we identified Exoc4, Cd36, Cxcl1/2–Cxcr2 signaling, and Sftpc (SP-C) as critical mediators of metastatic adaptation. Importantly, these results establish a hypoxia (HIf-2α)–Cd36–VM axis and reveal Sftpc (SP-C) as a lung-specific marker of metastatic TNBC, offering novel prognostic and therapeutic implications. Future studies dissecting the mechanistic underpinnings of these pathways and testing their therapeutic tractability may pave the way for precision strategies to prevent or treat TNBC lung metastasis.

## Supplementary information

Supplemental materials

The upregulated in WFDC12 subcluster

Western blot

Supplementary table3

## Data Availability

Requests for raw data or further information should be directed to and will be fulfilled by the corresponding author, Ya-Ling Hsu (yainghsu@kmu.edu.tw).
