# Supplementary material for: Single-cell and spatial transcriptomics map lung-adaptive metastasis programs through Exoc4, Cd36–vasculogenic mimicry, and Cxcr2 signaling in triple-negative breast cancer
Source: Cell Death Dis. 2026 Jul 7;17(1):846. doi: 10.1038/s41419-026-09049-6 (PMC13627727; doi:10.1038/s41419-026-09049-6)
Supplement: Supplementary file 1 [file 41419_2026_9049_MOESM1_ESM.pdf]

**Supplementary Figure legend**

**Supplementary Figure 1. Purity analysis of isolated 4T1-P, 4T1-L, MMTV-P, and MMTV-L cells.**

**Supplementary Figure 2. Additional spatial and functional validation of Exoc4 in TNBC lung metastasis.** (A) Spatial transcriptomic analysis confirming broad upregulation of the 24-gene C12-Cd metastatic signature in breast cancer lung metastatic lesions compared with the restricted expression pattern in the primary tumor. (B) Spatial analysis showing regional enrichment of Exoc4 expression in areas positive for the C12-Cd gene signature in primary and metastatic tissues. (C, D) Quantification of IHC Exoc4 staining in MMTV (C) and 4T1 (D) animal models. The efficiency of Exoc1 siRNA in both MMTV-L and 4T1-L cells. Significance: $p < 0.05$ (*), $p < 0.01$ (**), $p < 0.001$ (***), $p < 0.0001$ (****). NC, normal (Scramble) control RNA

**Supplementary Figure 3. The knockdown efficiency of Epas1 (Hif-2α) siRNA in both MMTV-P and 4T1-P cells.** Bars indicated mean ± SD. For two-group comparisons, unpaired Student's t-test was used. For comparisons involving more than two groups, one-way ANOVA with Tukey's post hoc test was used. Significance: $p < 0.05$ (*), $p < 0.01$ (**), $p < 0.001$ (***), $p < 0.0001$ (****).

**Supplementary Figure 4. Cd36 regulates the angiogenic capacity of TNBC cells**. (A) Tube formation assay showing that Cd36 overexpression significantly increased the angiogenic capacity of MMTV-P and 4T1-P cells. (B) Tube formation assay showing that Cd36 knockdown significantly decreased the angiogenic capacity of MMTV-L and 4T1-L cells.

**Supplementary Figure 5. Validation of Sftpc expression and knockdown efficiency.** (A–B) Quantitative analysis of IHC staining for SP-C in the MMTV (A) and 4T1 (B) animal models. (C) Validation of Sftpc knockdown efficiency in 4T1-L cells. Data are presented as mean ± SD. Statistical significance was determined by Student's t-test.

Significance: $p < 0.05$ (*), $p < 0.01$ (**), $p < 0.0001$ (****).

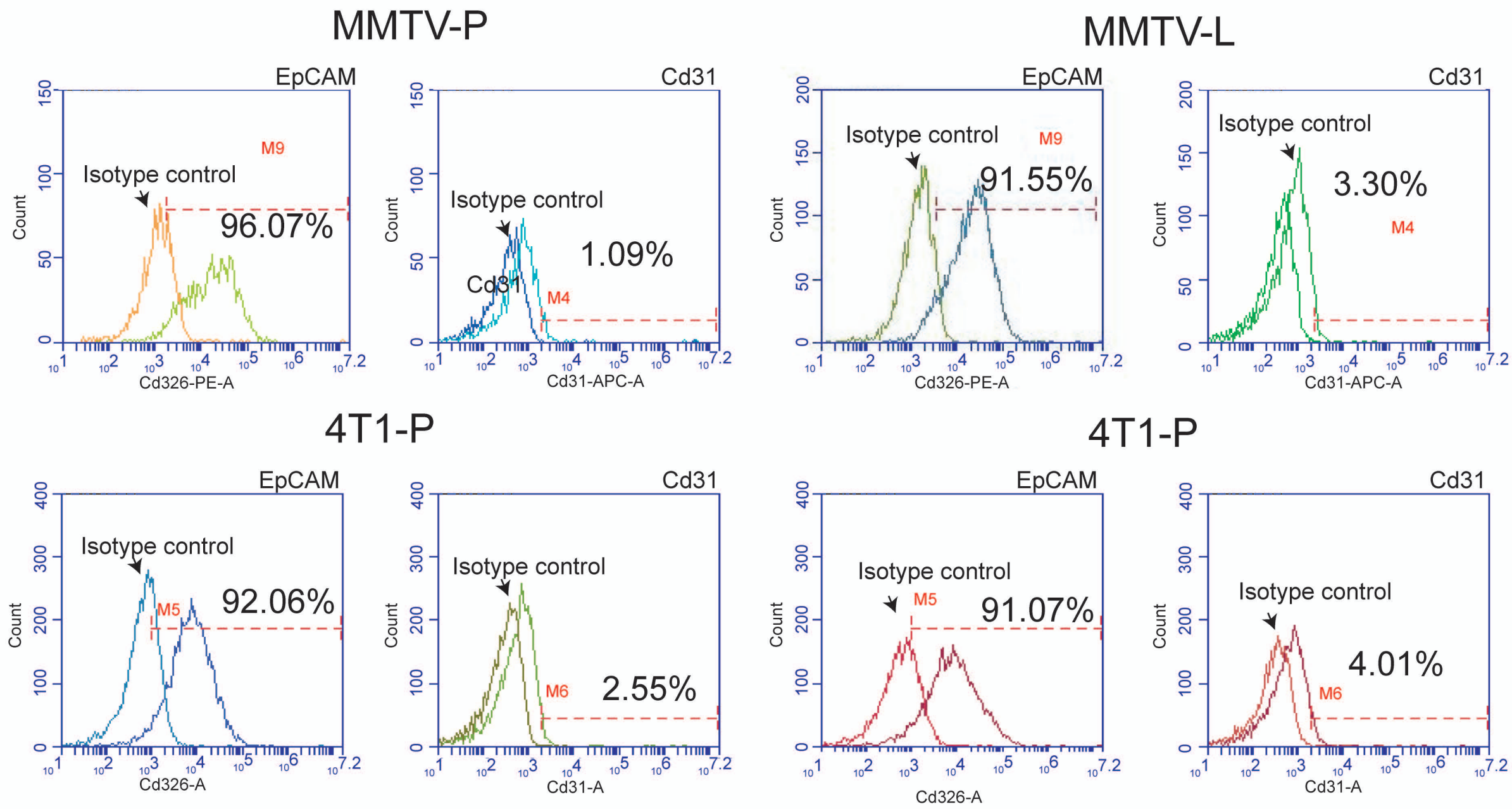

MMTV-P
MMTV-L
EpCAM
Cd31
Isotype control
96.07%
M9
Cd326-PE-A
1.09%
Cd31
M4
Cd31-APC-A
91.55%
3.30%
4T1-P
92.06%
M5
Cd326-A
2.55%
M6
Cd31-A
91.07%
4.01%
Count

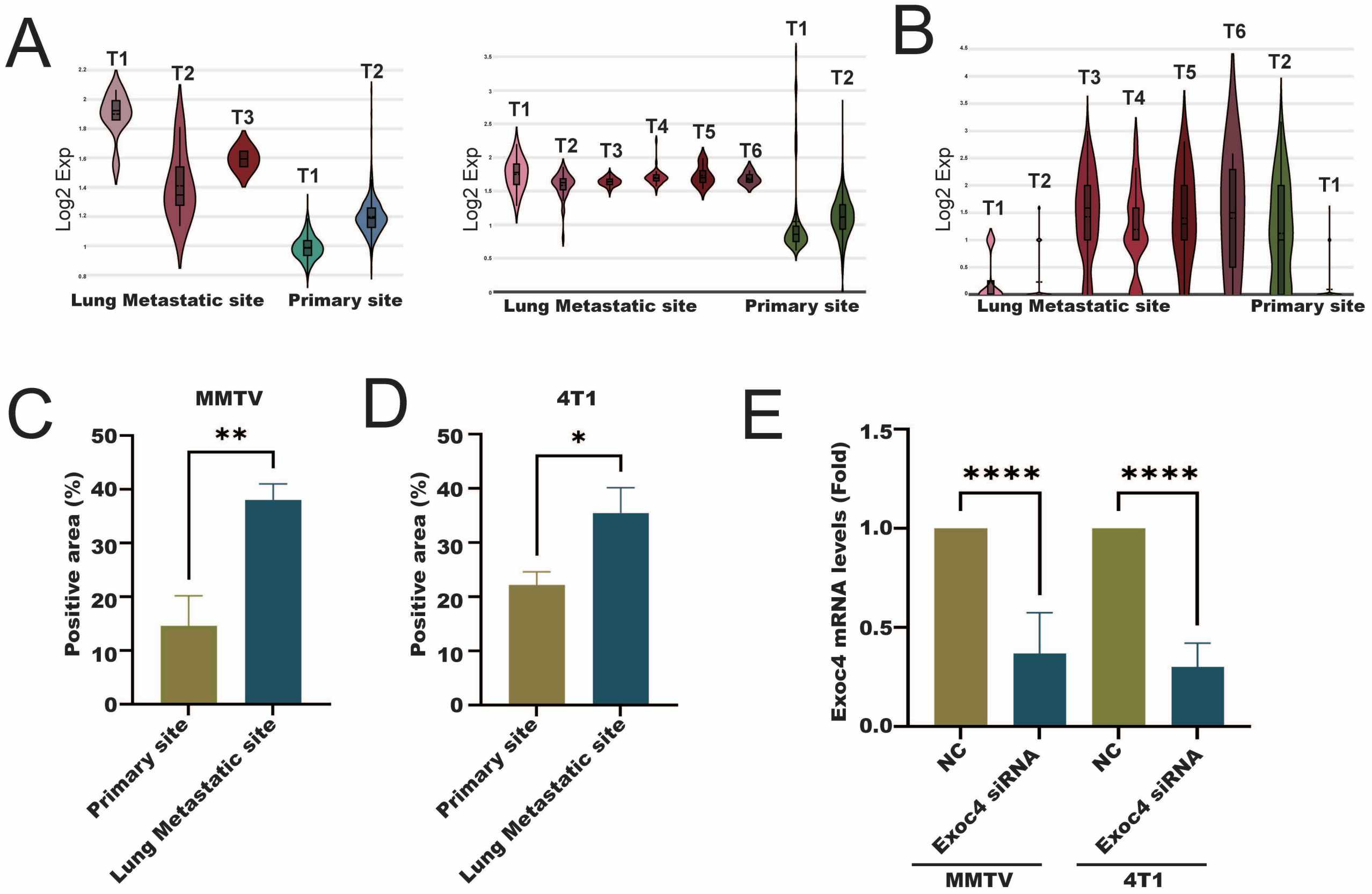

A
Log2 Exp
T1
T2
T3
T1
T2
Lung Metastatic site
Primary site
T1
T2
T3
T4
T5
T6
T1
T2
B
T1
T2
T3
T4
T5
T6
T2
T1
C
MMTV
Positive area (%)
**
Primary site
Lung Metastatic site
D
4T1
*
E
Exoc4 mRNA levels (Fold)
****
NC
Exoc4 siRNA
MMTV
4T1

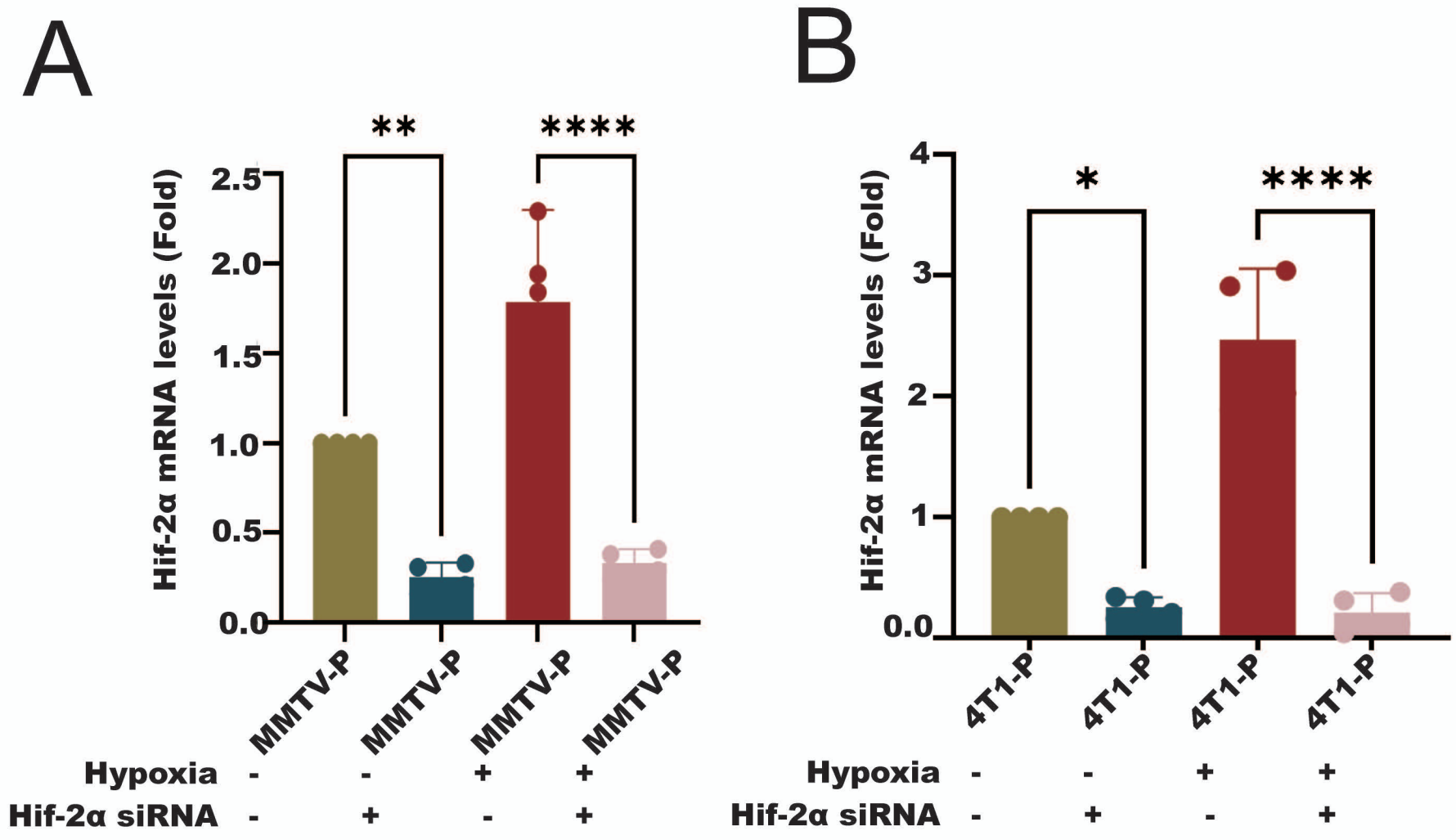
A
B
Hif-2α mRNA levels (Fold)
2.5
2.0
1.5
1.0
0.5
0.0
**
****
MMTV-P
MMTV-P
MMTV-P
MMTV-P
Hypoxia - - + +
Hif-2α siRNA - + - +
4
3
2
1
0.0
*
****
4T1-P
4T1-P
4T1-P
4T1-P
Hypoxia - - + +
Hif-2α siRNA - + - +

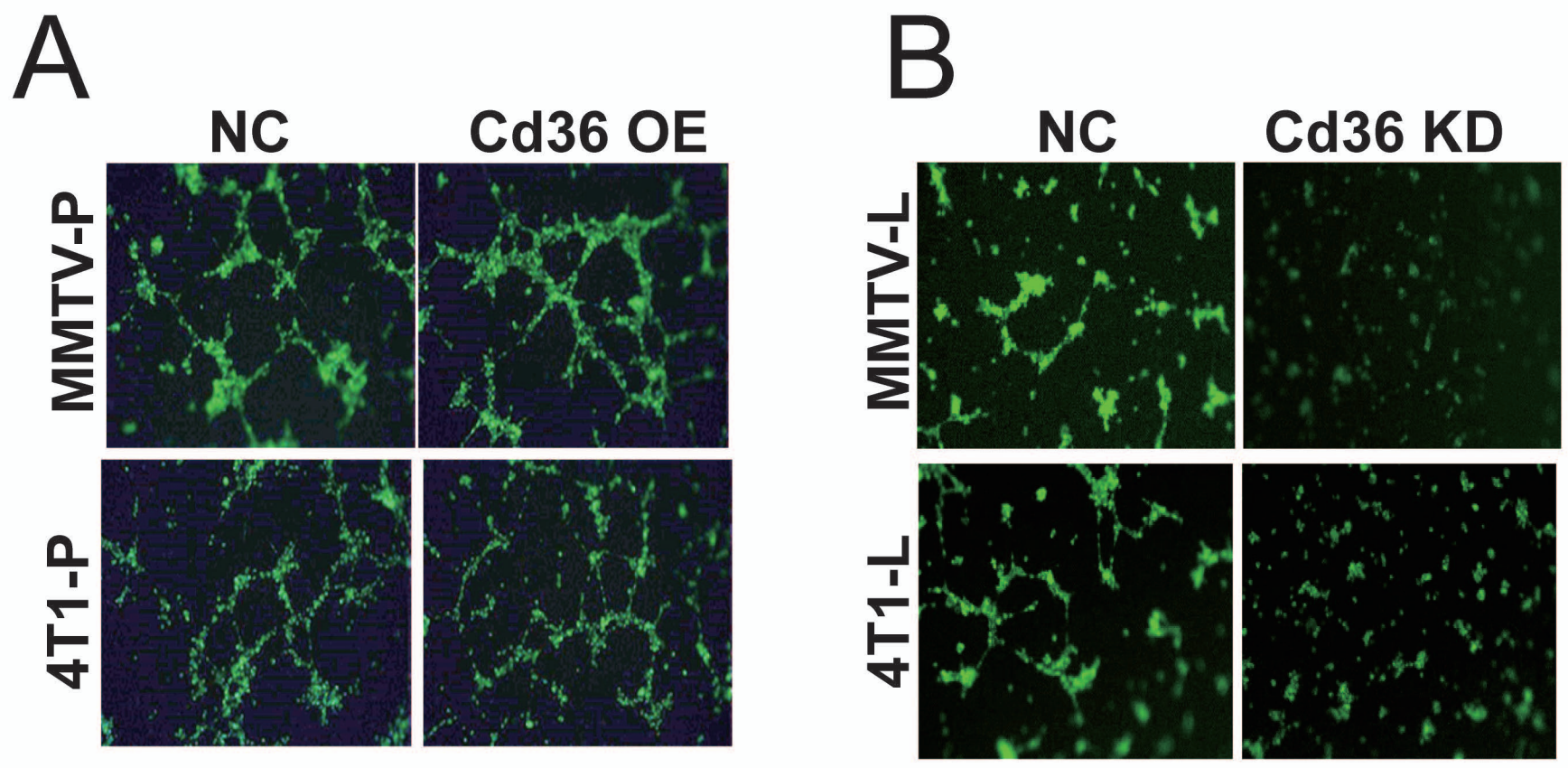
A
NC
Cd36 OE
MMTV-P
4T1-P
B
NC
Cd36 KD
MMTV-L
4T1-L

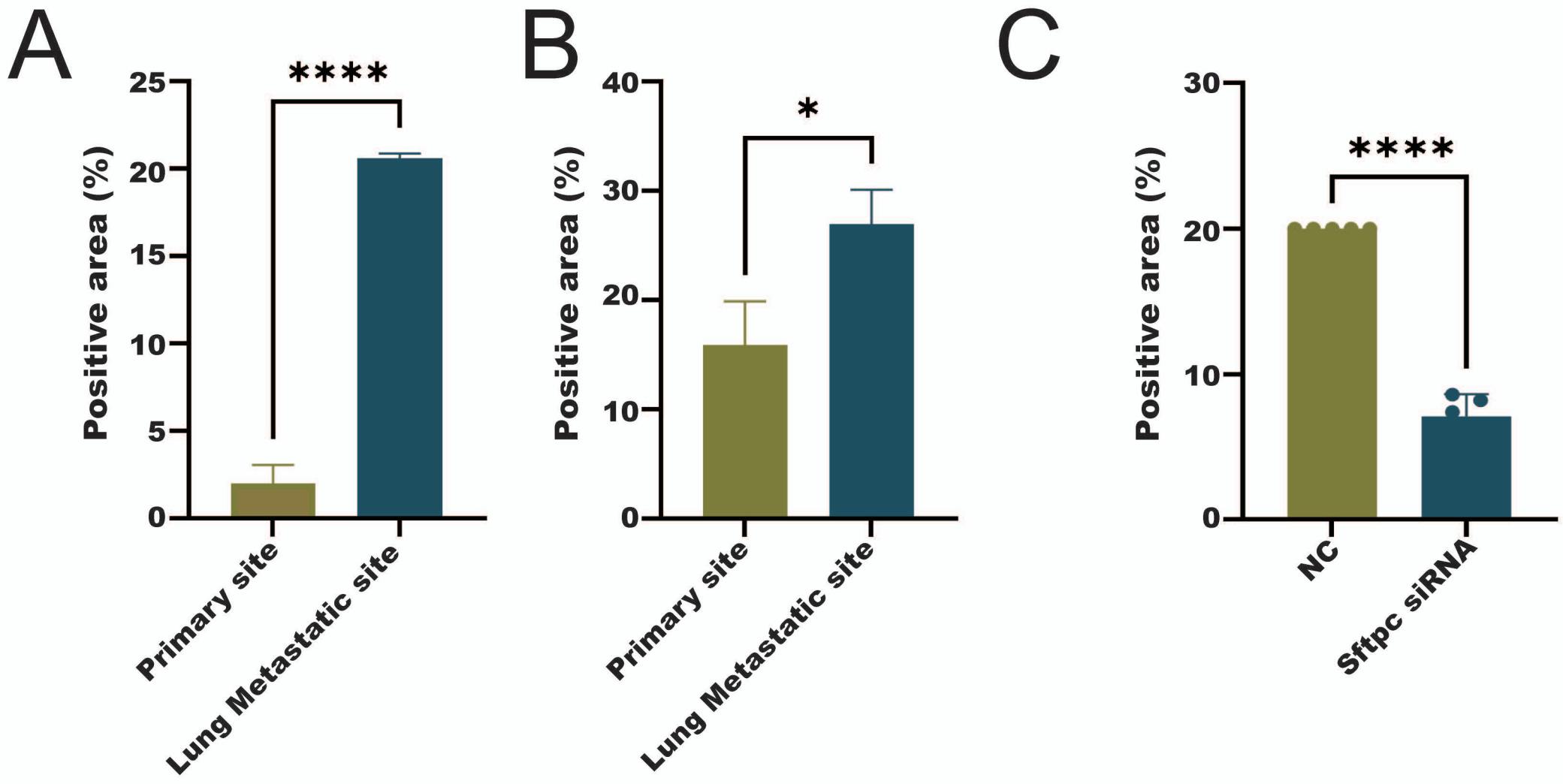

A
B
C
Positive area (%)
****
*
****
Primary site
Lung Metastatic site
NC
Sftpc siRNA
0
5
10
15
20
25
30
40

**sTable 1. The sequence of primers**

| | |
|---|---|
| *Exoc4* | F: AAG ATC CCT CTC CTG GTC CT |
| | R: TGT TCT CCT CTG GGT CAC AC |
| *Cldn10* | F: ATG TCC AGG GCA CAG ATC TC |
| | R: CAT GCC TGG ATG TAA CCT GC |
| *Etv1* | F: ACT GGA CGA GGC ATG GAA TT |
| | R: TTT CTC CAG CCA CCT TTT GC |
| *Zfp36* | F: GCT GTC ACC CTC ACC TAC TT |
| | R: TGT GGC AGA GTT CCG TTT TG |
| *Cdh5* | F: CTC ATC TCA GAC AAC GGG GT |
| | R: TGG TGA GGA TGC AGA GGA AG |
| *Epas1* | F: GCA AGC CTT CCA AGA CAC AA |
| | R: CCT GAG CTC CTG GTA GAT GG |
| *Sftpc* | F: GTG GTG ATT GTA GGG GCT CT |
| | R: CAG TGG AGC CGA TGG AAA AG |
| *Cd36* | F: GTG CAA AAC CCA GAT GAC GT |
| | R: TCC AAC AGA CAG TGA AGG CT |
| *Exoc4* | F: AAG ATC CCT CTC CTG GTC CT |
| | R:TGT TCT CCT CTG GGT CAC AC |
| *Gapdh* | F: AAC TTT GGC ATT GTG GAA GG |
| | R: ACA CAT TGG GGG TAG GAA CA |

**sTable 2. The antibodies used in this study**

| **Target** | **Brand** | **Category** | **Dilution power** |
|---|---|---|---|
| **Western blot** | | | |
| Hif-1α | Merck Millipore | MAB5382 | 1:1000 |
| Hif-2α | Cell signaling technology | 57921 | 1:1000 |
| E-cadherin | BD Transduction Laboratories™ | 610182 | 1:1000 |
| N-cadherin | BD Transduction Laboratories™ | 610921 | 1:1000 |
| Vimentin | BD Transduction Laboratories™ | 5550513 | 1:2000 |
| Akt | Cell signaling technology | 4691 | 1:1000 |
| Phospho-Akt | Cell signaling technology | 13038 | 1:1000 |
| Gapdh | Merck | MAB374 | 1:5000 |
| **IHC** | | | |
| Exoc4 | Proteintech | 11913-1-ap | 1:100 |
| Cd36 | Abcam | ab252923 | 1:50X |
| Cdh5 | Abcam | ab318152 | 1:100 |
| Cd31 | Abcam | ab28364 | 1:50X |
| SP-C | Abcam | ab90716 | 1:100X |
| **Flowcytometry** | | | |
| Isotype control | BD Pharmingen | 562141 | 0.02mg/ml |
| Cd36 | BD Pharmingen | 562702 | 0.02mg/ml |
| Isotype control | BD Pharmingen | 553932 | 0.02mg/ml |
| Cd31 | BD Pharmingen | 551262 | 0.02mg/ml |
| Isotype control | BD Pharmingen | 554689 | 0.02mg/ml |
| Cd326 | BD Pharmingen | 563477 | 0.02mg/ml |
