# Supplementary material for: Single-cell and spatial transcriptomics map lung-adaptive metastasis programs through Exoc4, Cd36–vasculogenic mimicry, and Cxcr2 signaling in triple-negative breast cancer
Source: Cell Death Dis. 2026 Jul 7;17(1):846. doi: 10.1038/s41419-026-09049-6 (PMC13627727; doi:10.1038/s41419-026-09049-6)
Supplement: Supplementary file 2 [file 41419_2026_9049_MOESM3_ESM.pdf]

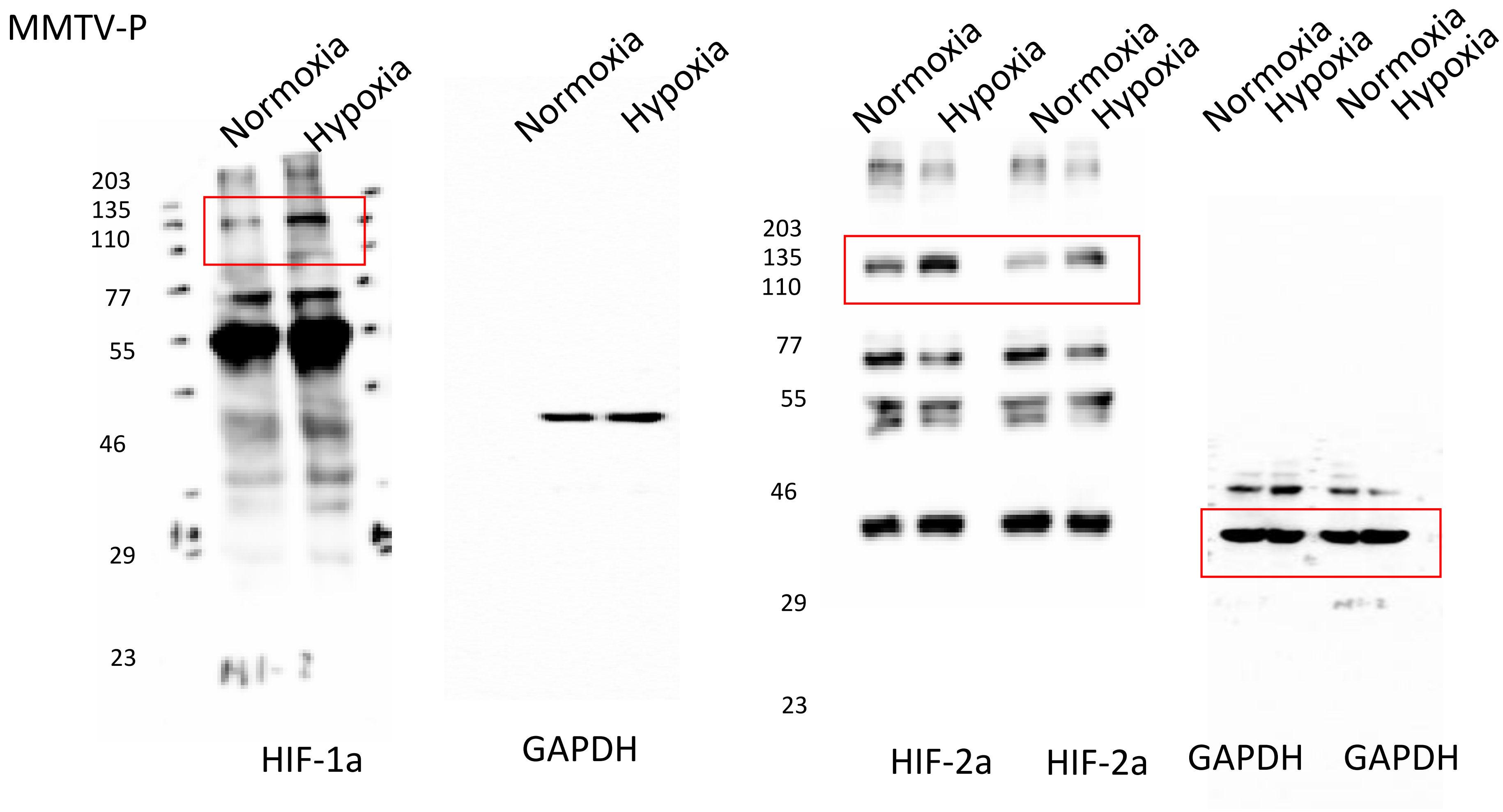
MMTV-P
Normoxia
Hypoxia
Normoxia
Hypoxia
203
135
110
77
55
46
29
23
HIF-1a
GAPDH
Normoxia
Hypoxia
Normoxia
Hypoxia
Normoxia
Hypoxia
Normoxia
Hypoxia
203
135
110
77
55
46
29
23
HIF-2a
HIF-2a
GAPDH
GAPDH

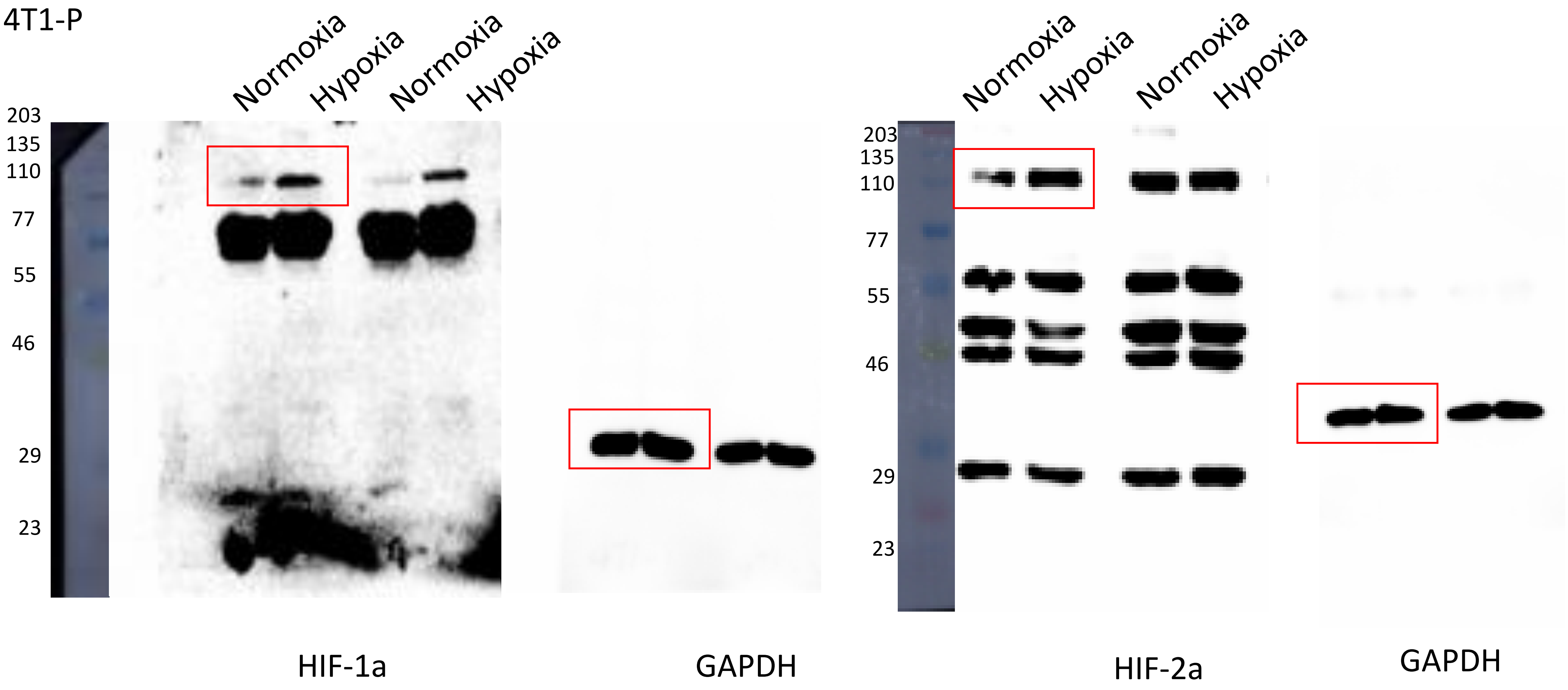
4T1-P
Normoxia
Hypoxia
Normoxia
Hypoxia
203
135
110
77
55
46
29
23
Normoxia
Hypoxia
Normoxia
Hypoxia
203
135
110
77
55
46
29
23
HIF-1a
GAPDH
HIF-2a
GAPDH

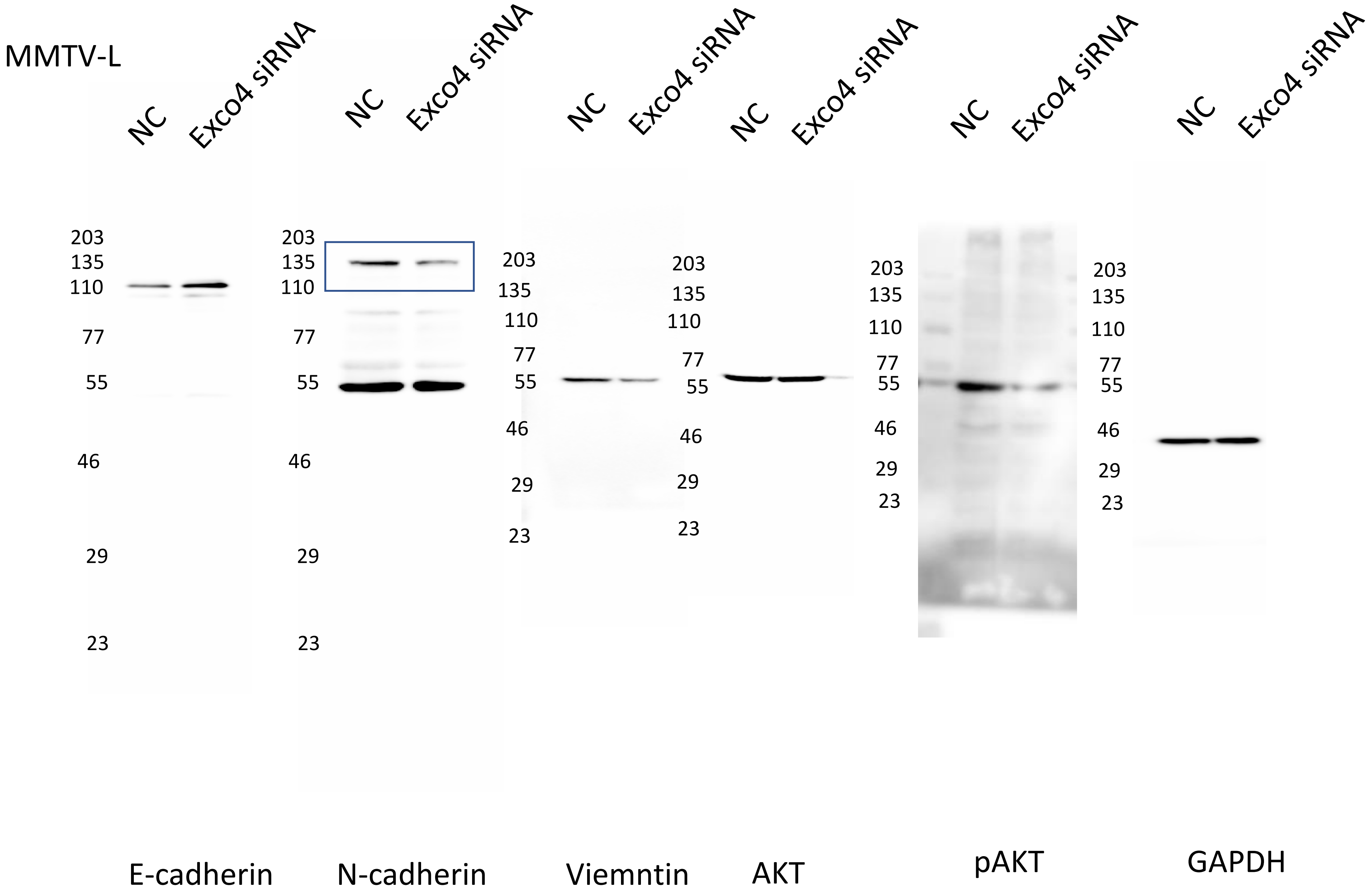
MMTV-L
NC
Exco4 siRNA
203
135
110
77
55
46
29
23
E-cadherin
N-cadherin
Viemntin
AKT
pAKT
GAPDH

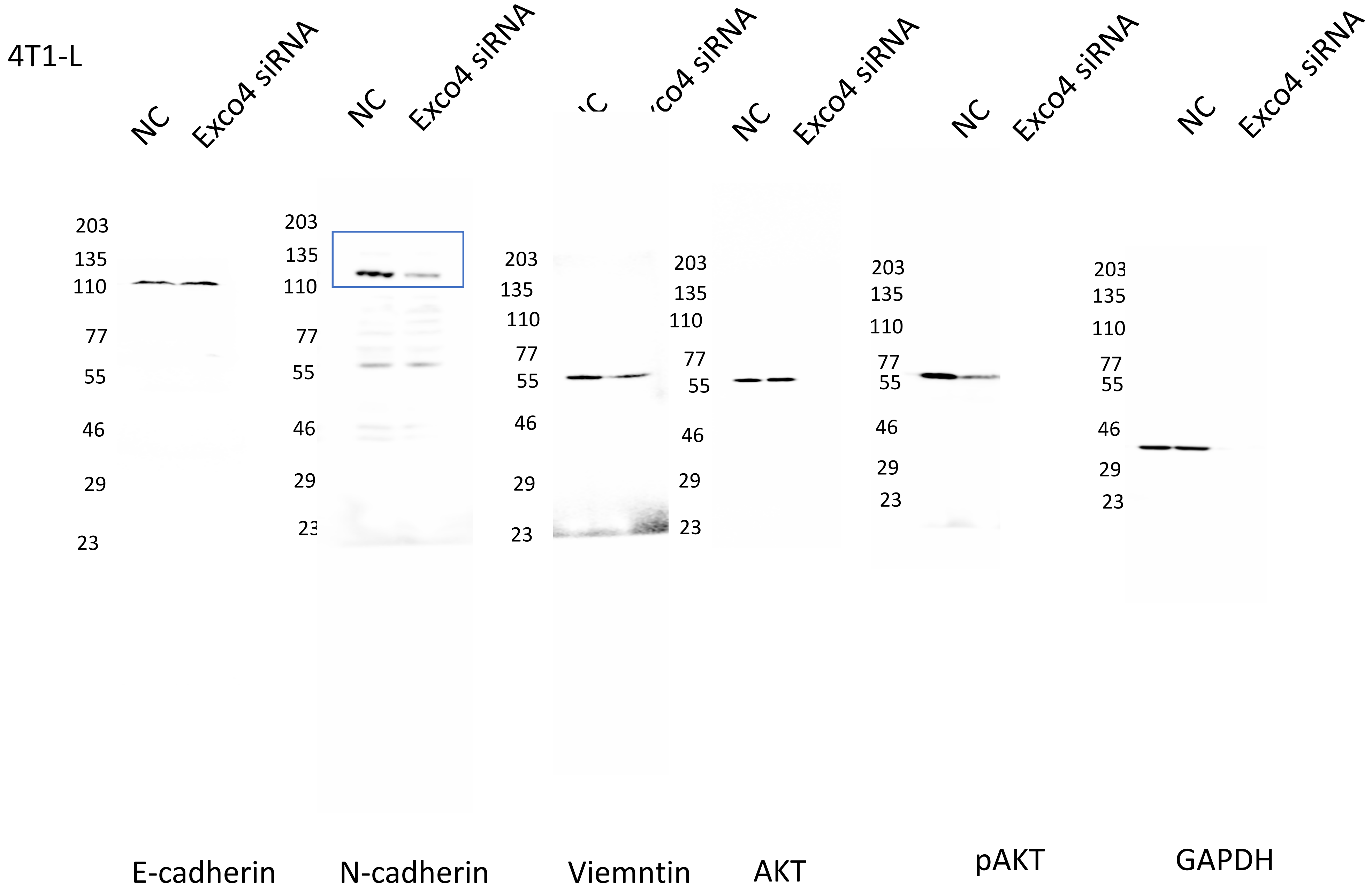
4T1-L
NC
Exco4 siRNA
203
135
110
77
55
46
29
23
E-cadherin
N-cadherin
Viemntin
AKT
pAKT
GAPDH
